# Evolution of Phototrophy in the Chloroflexi Phylum Driven by Horizontal Gene Transfer

**DOI:** 10.3389/fmicb.2018.00260

**Published:** 2018-02-19

**Authors:** Lewis M. Ward, James Hemp, Patrick M. Shih, Shawn E. McGlynn, Woodward W. Fischer

**Affiliations:** ^1^Division of Geological and Planetary Sciences, California Institute of Technology, Pasadena, CA, United States; ^2^Department of Gastroenterology, University of Utah School of Medicine, Salt Lake City, UT, United States; ^3^Department of Energy, Joint BioEnergy Institute, Emeryville, CA, United States; ^4^Environmental Genomics and Systems Biology Division, Lawrence Berkeley National Laboratory, Berkeley, CA, United States; ^5^Earth-Life Science Institute, Tokyo Institute of Technology, Meguro, Japan

**Keywords:** lateral gene transfer, comparative genomics, microbial metabolism, phylogenetics, microbial diversity

## Abstract

The evolutionary mechanisms behind the extant distribution of photosynthesis is a point of substantial contention. Hypotheses range from the presence of phototrophy in the last universal common ancestor and massive gene loss in most lineages, to a later origin in Cyanobacteria followed by extensive horizontal gene transfer into the extant phototrophic clades, with intermediate scenarios that incorporate aspects of both end-members. Here, we report draft genomes of 11 Chloroflexi: the phototrophic Chloroflexia isolate *Kouleothrix aurantiaca* as well as 10 genome bins recovered from metagenomic sequencing of microbial mats found in Japanese hot springs. Two of these metagenome bins encode photrophic reaction centers and several of these bins form a metabolically diverse, monophyletic clade sister to the Anaerolineae class that we term *Candidatus* Thermofonsia. Comparisons of organismal (based on conserved ribosomal) and phototrophy (reaction center and bacteriochlorophyll synthesis) protein phylogenies throughout the Chloroflexi demonstrate that two new lineages acquired phototrophy independently via horizontal gene transfer (HGT) from different ancestral donors within the classically phototrophic Chloroflexia class. These results illustrate a complex history of phototrophy within this group, with metabolic innovation tied to HGT. These observations do not support simple hypotheses for the evolution of photosynthesis that require massive character loss from many clades; rather, HGT appears to be the defining mechanic for the distribution of phototrophy in many of the extant clades in which it appears.

## Introduction

Multiple hypotheses exist for the origin and subsequent evolution of photosynthesis, but little is known with certainty. It is widely held that anoxygenic preceded oxygenic photosynthesis, but which of the extant taxa—if any—invented phototrophy and/or were phototrophic progenitors on the early Earth remains unclear (Fischer et al., 2016). While simple forms of photoheterotrophy can be driven by proton-pumping rhodopsins, light-driven electron transport—and therefore the possibility of light-driven carbon fixation (i.e., photosynthesis)—is only known to be driven by organisms utilizing phototrophic reaction centers. Here we focus on reaction center-based phototrophy, as it can drive electron transport and therefore photosynthesis, and was responsible for major environmental transitions through Earth history (Fischer et al., 2016; Ward, 2017). To date, reaction center-based phototrophy has been identified in seven bacterial phyla—the Cyanobacteria, Chlorobi, Chloroflexi, Acidobacteria, Firmicutes, Gemmatimonadetes, and Proteobacteria. Of these, only one—the Cyanobacteria—contains members that possess two photosystems, coupled in series to perform oxygenic photosynthesis. The others perform anoxygenic phototrophy, and possess only a single reaction center, either of the Type 1 (Chlorobi, Heliobacteria, and Acidobacteria) or Type 2 (Proteobacteria, Gemmatimonadetes, and Chloroflexi) variety. It was hypothesized that photosynthesis was present in the last common ancestor of all bacteria (Woese et al., 1985; Woese, 1987) or a broad clade containing all extant phototrophs (Cardona, 2016), followed by extensive loss in most lineages; however, this idea remains controversial. The distribution of phototrophy across the bacterial tree is sparse, with phototrophic clades scattered across the domain rather than forming a single clade of phototrophs. Even Type 1- and Type 2-reaction center bearing phototrophs are mixed (e.g., the closest phototrophic relative of the phototrophic Chlorobi are phototrophic Gemmatimonadetes; the former has a Type 1 reaction center and the latter a Type 2—a relationship inconsistent with vertical inheritance; Fischer et al., 2016). This pattern suggests instead an alternative scenario involving a later origin of phototrophy (sometime after the origin of the bacterial domain), followed by multiple instances of horizontal gene transfer (HGT) that resulted in the modern distribution of phototrophy (e.g., Igarashi et al., 2001; Raymond et al., 2002; Hohmann-Marriott and Blankenship, 2011; Nagashima and Nagashima, 2013; Zeng et al., 2014; Fischer et al., 2016).

The most straightforward tests of these hypotheses arise by comparing the organismal phylogenies of phototrophic bacteria to phylogenies of photosynthesis genes—concordance of the trees would be consistent with shared ancestry, while discrepancies between them would indicate a history of horizontal gene transfer (Doolittle, 1986). While the structure of the bacterial tree of life is still debated (e.g., Woese, 1987; Williams et al., 2013; McInerney et al., 2014; Hug et al., 2016; Schulz et al., 2017), intra-phylum organismal relationships tend to be robust (e.g., reproduced via many different markers) despite enduring uncertainty in relationships between phyla (Pace, 2009). As a result, the history of metabolic characters like photosynthesis within a phylum is more straightforward to assess than it is for the bacteria as a whole. If a major role for horizontal gene transfer can be demonstrated within a particular phylum, the HGT-driven phototrophy hypothesis will be strengthened, whereas a concordance of organismal and gene trees would be more consistent with an ancient origin and vertical inheritance of the metabolism. While tests of this kind have been made previously in the Proteobacteria, suggesting intra-phylum horizontal gene transfer (Igarashi et al., 2001; Swingley et al., 2009; Nagashima and Nagashima, 2013), this has not previously been possible in other phototrophic phyla due to the limited diversity of phototrophic members within each. However, the discovery of new phototrophic lineages via metagenomic sequencing provides opportunities for querying the evolutionary history of phototrophs. In particular, the recent description of *Candidatus* Roseilinea gracile—a phototrophic Chloroflexi closely related to the non-phototrophic Anaerolineae class and only distantly related to known phototrophic Chloroflexi in the Chloroflexia class (Klatt et al., 2011; Thiel et al., 2016, 2017; Tank et al., 2017)—suggests that the diversity and evolutionary history of phototrophy in the Chloroflexi is richer than previously thought.

**Figure 1:**
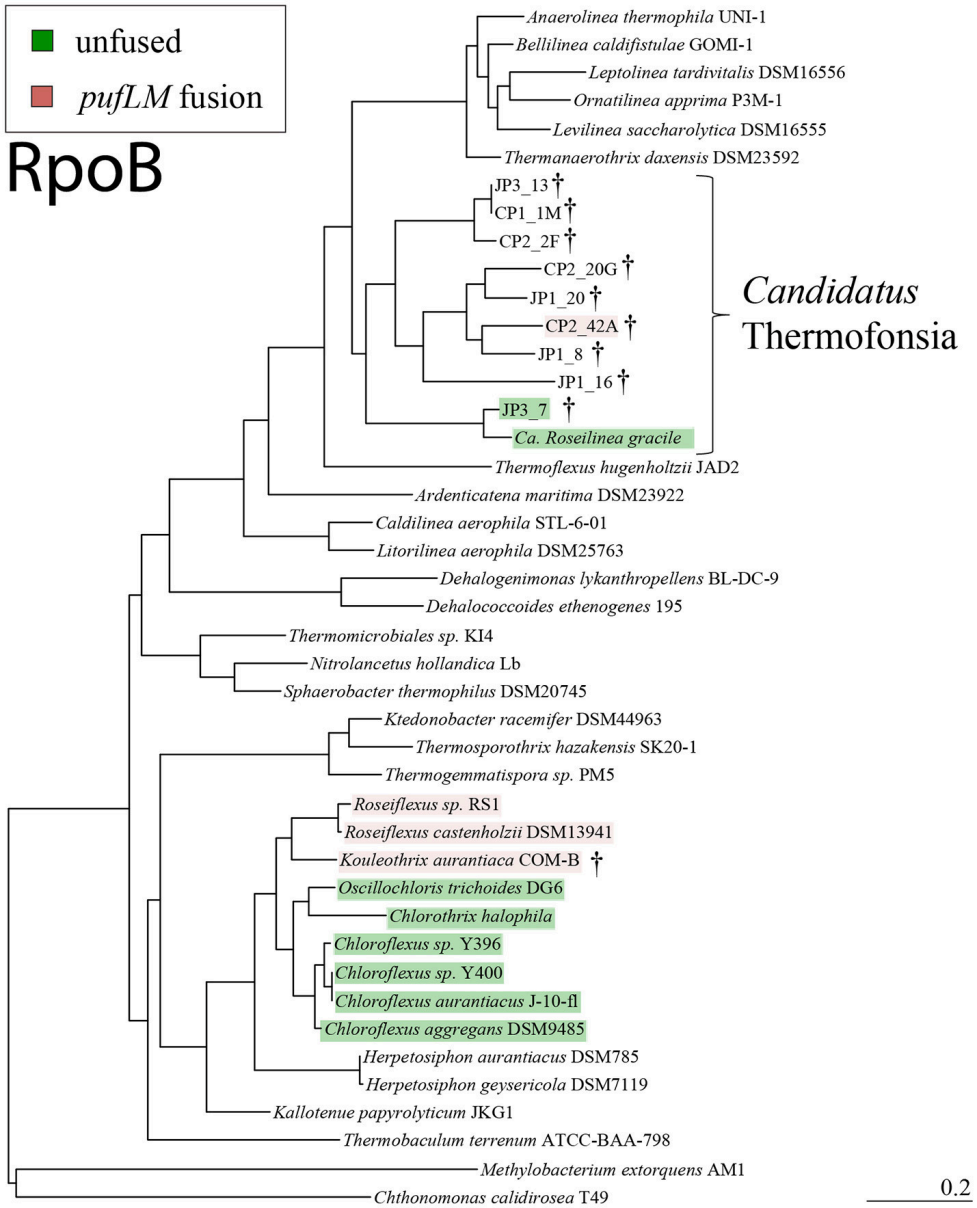
Reference phylogeny of Chloroflexi based on RpoB protein sequences, with our newly sequenced strains indicated with daggers, phototrophic strains highlighted (pink for fused *pufLM*, green for unfused), and *Candidatus* Thermofonsia noted. Most phototrophic Chloroflexi occur within a monophyletic clade in the Chloroflexia class, yet two distinct lineages of phototrophs occur outside of this class, separated by many non-phototrophic lineages. This phylogeny is based on RpoB—a single organismal marker protein which was recovered in all *Ca*. Thermofonsia genome bins—and is primarily intended as a reference for the critical phylogenetic relationships presented here (e.g., divergence of *Ca*. Thermofonsia from Anaerolineae, separation of phototrophic Thermofonsia from phototrophic Chloroflexia). Potentially more robust organismal phylogenies (e.g., 16S or larger concatenated protein datasets) will be possible with higher completeness *Ca*. Thermofonsia genomes.

The Chloroflexi (e.g., Green Non-sulfur Bacteria) are a phylum of primarily gliding, filamentous bacteria possessing a wide diversity of metabolisms and ecological roles, but are best known as photoheterotrophs (Overmann, 2008). Chloroflexi are notably abundant in a range of environments (e.g., marine sediments and groundwater, Inagaki et al., 2003; Hug et al., 2013). Despite their environmental richness revealed by culture-independent surveys, most well-characterized Chloroflexi belong to a few subclades isolated from hot springs (Yamada and Sekiguchi, 2009), including the anoxygenic phototrophic *Chloroflexus* (Pierson and Castenholz, 1974; Hanada et al., 1995) and *Roseiflexus* (Hanada et al., 2002). Based on phylogenetic analysis of chlorophyll and bacteriochlorophyll synthesis genes, it was suggested that anoxygenic phototrophy in this group predates the evolution of oxygenic photosynthesis in Cyanobacteria (Xiong et al., 2000); if correct it would imply that this group is remarkably ancient, and therefore might provide a window into phototrophy on the early Earth. Recent genomic sequencing projects have expanded the known taxonomic and metabolic diversity of the Chloroflexi phylum (e.g., the Ardenticatenia class, capable of nitrate- and iron oxide- reduction, Kawaichi et al., 2013, 2015; Hemp et al., 2015b). Newly discovered Chloroflexi are diverse in terms of morphology, metabolism, and other traits (Table 1), but continue to be recovered as a monophyletic clade in phylogenetic trees (Figure 1) and have sufficient sequence similarity to be classified as a single phylum (Hanada, 2014).

**Table 1:** Characteristics of Chloroflexi classes.

	**Chloroflexia^a^**	**Thermomicrobia^b^**	**Anaerolineae^c^**	**Caldilineae^d^**	**Ardenticatenia^e^**	**Ktedenobacteria^f^**	**Thermoflexia^g^**	**Dehalococcoidetes^h^**	***Ca*. Thermofonsia^i^**
Phototrophy	+ (reaction center-based)	–	–	–	–	–	–	–	Some (reaction center- or rhodopsin-based)
Aerobic respiration	+	+	Genes present	Genes present	+	+	Microaerophilic	Genes rarely present	Genes present
Complex III	bc, ACIII, or both	ACIII or both	ACIII, bc, or neither	bc	bc	bc	bc	Neither	bc, also ACIII with RCII
Morphology	Filamentous	Rod	Filamentous or rods	Filamentous	Filamentous	Filamentous (branched)	Filamentous	Coccoidal, discs	Unknown
Motility	Gliding	Flagellar	Flagellar or none	–	–	–	–	–	Unknown—no flagellar genes
Other metabolic traits		Nitrite oxidation			Iron and nitrogen respiration			Dehalogenation	Nitrogen respiration
Temperature range	10–67	43–80	20–73	37–65	30–75	17–74	67.5–75	15–35	32–59
% GC	48–62	56–63	48–58	59–65	51.5	54–60	69	49–54	46–63

a *(Garrity and Holt, 2001; Gupta et al., 2013)*.

b *(Hugenholtz and Stackebrandt, 2004; Sorokin et al., 2012)*.

c *(Yamada et al., 2006; Hemp et al., 2015a,b,c; Pace et al., 2015; Ward et al., 2015a,b)*.

d *(Yamada et al., 2006)*.

e *(Kawaichi et al., 2013)*.

f *(Cavaletti et al., 2006; Yabe et al., 2010; Chang et al., 2011)*.

g *(Dodsworth et al., 2014)*.

h *(Moe et al., 2009; Löffler et al., 2013)*.

i *This study*.

Here, we report eleven draft Chloroflexi genomes: ten recovered from hot spring microbial mats in Japan as well as one previously cultured isolate. These genomes include two new phototrophs outside of the classically phototrophic Chloroflexia class, as well as several members of a novel class-level clade sister to the Anaerolinea. Distinct phylogenetic patterns of organismal and phototrophic proteins demonstrate the role of horizontal gene transfer during the evolution of phototrophy within this phylum.

## Materials and methods

### Genomic sequencing of *Kouleothrix aurantiaca*

The genome of *K. aurantiaca* COM-B (JCM 19913) was sequenced as part of a project to expand the phylogenetic breadth of Chloroflexi genomes (Hemp et al., 2015a,b,c; Pace et al., 2015; Ward et al., 2015a,b). *K. aurantiaca*, a member of the group formerly known as ‘Eikenboom morphotype 1851’ (Seviour and Blackall, 1999), was isolated from activated sludge in an industrial wastewater treatment facility (Kohno et al., 2002). It forms orange-pigmented cells organized into long mm-scale filaments, grows on pyruvate and by fermentation of certain sugars, and can reduce nitrate to nitrite (Kohno et al., 2002). It is closely related to members of the genus *Roseiflexus* (Beer et al., 2002), however phototrophy has not been observed in these organisms in culture.

Genome sequencing was performed at Seqmatic (Fremont, CA) using the Illumina MiSeq sequencing platform. SPAdes 3.1.1 (Nurk et al., 2013) was used to assemble the genome. The genome was screened for contaminants based on sequence coverage, GC composition, and BLAST hits of conserved single copy genes. Genome annotation was performed using the NCBI Prokaryotic Genome Annotation Pipeline.

### Metagenomic sample collection

Four metagenomic datasets were recovered from two hot springs in Japan: Jinata Onsen and Nakabusa Onsen (Supplemental Table 1). Genome bins labeled JP1 or JP3 were derived from Jinata Onsen, while CP1 and CP2 were derived from Nakabusa Onsen (Table 2).

**Table 2:** Genome statistics of sequenced strains.

	**Genome size**	**% GC**	**No. coding sequences**	**No. Contigs**	**Completeness**	**Contamination**	**Strain Heterogeneity**	**tRNAs**	**Source**
CP1_1M	1.39	59	1,182	138	42.28	1.81	50	14	Nakabusa Cone Pool 1
CP2_2F	1.99	59	1,734	20	49.46	0	0	23	Nakabusa Cone Pool 2
CP2_20G	3.09	48	2,678	852	78.54	3.55	33.33	32	Nakabusa Cone Pool 2
CP2_42A	3.3	59	2,897	2,024	79.44	10.42	16.13	31	Nakabusa Cone Pool 2
JP1_8	2.21	51	1,973	601	58.13	0.13	0	17	Jinata Pool 1
JP1_16	4.06	44	3,238	1,764	95.15	17.31	0	45	Jinata Pool 1
JP1_20	3.36	46	2,878	1,139	79.09	4.78	20	34	Jinata Pool 1
JP1_191	0.417	47	334	883	10.63	1.8	0	7	Jinata Pool 1
JP3_7	3.62	63	3,078	1,331	87	12.85	7.32	46	Jinata Pool 3
JP3_13	3.67	60	3,116	1,259	96.17	10.87	75	46	Jinata Pool 3
*Kouleothrix aurantiaca*	8.7	62	8,993	5,539	85	0	0	97	Isolate from wastewater sludge

Jinata genome bins were assembled from two metagenomes from Jinata Onsen, on Shikinejima Island, Tokyo Prefecture at 34.326111N, 139.216E. The geochemistry and microbial diversity of this spring were described in detail elsewhere (Ward, 2017). Shikinejima is part of the Izu Islands, a chain of volcanic islands that formed in the past 2-3 million years along the northern edge of the Izu-Bonin-Mariana Arc (Kaneoka et al., 1970). The source water of Jinata Onsen emerges anoxic, iron-rich, and gently bubbling from the spring source (Supplemental Figure 1). Temperature at the source was ~62°C. This spring water flows into a series of pools that mix progressively more with seawater during high tide, creating a range of geochemical conditions over short spatial and temporal scales as hot, iron-rich, oxygen-poor spring water mixes with cold, oxygen-rich seawater. The metagenomes from which JP1 bins were sequenced came from samples of thin (~1 mm) microbial mats in an iron-oxide rich pool near the spring source (Pool 1), while JP3 genomes were recovered from a Cyanobacteria-rich microbial mat in Pool 3, the most downstream section of the hot spring before it flows into the coastal ocean. Dissolved oxygen (DO), pH, and temperature measurements were performed *in situ* using an Exetech DO700 8-in-1 Portable Dissolved Oxygen Meter. Iron concentrations were measured using the ferrozine assay (Stookey, 1970) following acidification with 40 mM sulfamic acid to inhibit iron oxidation by O_2_ or oxidized nitrogen species (Klueglein and Kappler, 2013). At the time of sampling, Pool 1 was 59°C, pH 5.8, contained 1.8 mg/L DO and 265 μM Fe^2+^; Pool 3 was 46°C, pH 6.7, and contained 5.6 mg/L DO and 100 μM Fe^2+^.

Nakabusa genome bins were assembled from two metagenome samples collected from microbial mats from Nakabusa Onsen, located at 36.392429N, 137.748038E in the Japanese Alps near Azumino, Nagano Prefecture. Geochemical and microbiological characterization of the sampling site at Nakabusa Onsen is described in detail elsewhere (Kubo et al., 2011; Everroad et al., 2012; Otaki et al., 2012; Ward, 2017). Nakabusa Onsen is a sulfidic, moderately alkaline hot spring with source waters near 70°C (Supplemental Figure 2). The samples from which the metagenomes were sequenced were of cone-forming microbial mats at two points along the outflow from the hot spring source; Cone Pool 1 (the source of CP1 genomes) was a Chloroflexi-dominated mat located near the hot spring source, which at the time of sampling was 48°C and pH 8.1, while Cone Pool 2 (the source of the CP2 genomes) was collected from a cone-forming, Cyanobacteria-rich microbial mat several meters downstream, which at the time of sampling was 32°C and pH 8.3.

Samples of microbial mats were collected using sterile forceps and spatulas (~0.25 cm^3^ of material). Cells were lysed and DNA preserved in the field using Zymo Terralyzer BashingBead Matrix and Xpedition Lysis Buffer (Zymo Research, Irvine, CA). Cells were disrupted immediately by attaching tubes to the blade of a cordless reciprocating saw (Black & Decker, Towson, MD) and operating for 1 min.

### Metagenomic sequencing and analysis

Following return to the laboratory, DNA was extracted and purified with a Zymo Soil/Fecal DNA extraction kit (Zymo Research, Irvine, CA). DNA was quantified with a Qubit 3.0 fluorimeter (Life Technologies, Carlsbad, CA) according to manufacturer's instructions following DNA extraction. Purified DNA was submitted to SeqMatic LLC (Fremont, CA) for library preparation and 2 × 100 bp paired-end sequencing via Illumina HiSeq 4,000 technology. Raw sequence reads were assembled with MegaHit v. 1.02 (Li et al., 2016) and genome bins constructed based on tetranucleotide frequency using MetaWatt version 3.5.2 (Strous et al., 2012). Genomes were manually screened for genes of interest and uploaded to RAST (Aziz et al., 2008) for overall characterization. Genome bins were assessed for completeness and contamination using CheckM (Parks et al., 2015). Genes of interest (e.g., coding for ribosomal, photosynthesis, and electron transport proteins) were screened against outlier (e.g., likely contaminant) contigs as determined by CheckM using tetranucleotide, GC, and coding density content.

### Phylogenetics

Sequences of ribosomal and phototrophy proteins used in analyses (see below) were identified locally with the tblastn function of BLAST+ (Camacho et al., 2008), aligned with MUSCLE (Edgar, 2004), and manually curated in Jalview (Waterhouse et al., 2009). Positive BLAST hits were considered to be full length (e.g., >90% the shortest reference sequence from an isolate genome) with e values greater than 1e-20. Phylogenies were constructed using translated protein sequences. Phylogenetic trees were calculated using RAxML (Stamatakis, 2014) on the Cipres science gateway (Miller et al., 2010). Trees were visualized with SeaView (Gouy et al., 2010) and the Interactive Tree of Life viewer (Letunic and Bork, 2016).

### Probability of missing genes

In order to estimate the probability that certain sets of genes were missing from recovered genome bins, we calculated the probability mass function of recovering zero genes of a particular set from a genome of predicted size, given estimated completeness and assuming random sampling without replacement of individual genes. Though gene size varies significantly and colocalization makes selection of related genes not entirely independent, we assumed here that all genes have an equal probability of being selected. This simplifying assumption is reasonable, as recovered phototrophy genes largely reside on separate contigs (suggesting that colocalization is limited—in contrast to phototrophic Proteobacteria and Gemmatimonadetes, e.g., Igarashi et al., 2001; Nagashima and Nagashima, 2013; Zeng et al., 2014)—and the length of phototrophy-related genes (e.g., coding for reaction center proteins, bacteriochlorophyll synthases, etc.) are within error of average gene length. The calculation took the form of $f\left(x\right)=\left(\begin{array}{c} n\\ x \end{array}\right)\;\left(\begin{array}{c} T-n\\ r-x \end{array}\right)/\left(\begin{array}{c} T\\ r \end{array}\right)$, where *f* is the probability of recovering *x* genes of set *r* from a genome containing *T* genes of which *n* are recovered. In the case of our genome bins, *n* equaled the number of protein coding sequences recovered in each bin, *T* equaled *n* divided by the completeness of the genome as estimated by CheckM, and *r* equaled 6 (representing *pufL, pufM, pufC, bchX, bchY*, and *bchZ*). The probability that phototrophy genes existed in *Ca*. Thermofonsia genomes, but was not recovered in our bins, ranged from ~0.5 for JP1_191 (at only ~10% completeness) to ~2 × 10^−13^ for JP3_13 (at over 96% completeness). The probability of missing phototrophy genes was only >5% in JP1_191, greatly improving confidence that the absence of phototrophy from most strains of *Ca*. Thermofonsia is a real signal and not due to incomplete genomic data.

A similar calculation can be made for the probability that *bchL, bchN, bchB, bchM*, or *bchE* genes are present in phototrophic Thermofonsia, but simply not recovered in the genome bins. The probability of missing all five of these genes is about 0.03% for CP2_42A and less than 0.005% for JP3_7. It is therefore statistically likely that several (or all) of the missing bacteriochlorophyll synthesis genes are indeed missing from the genomes of phototrophic Thermofonsia, where the bacteriochlorophyll synthesis functions of these genes are potentially replaced by promiscuous homologs or other proteins.

A complementary analysis of the probability of false positives can be made to quantify the likelihood that all genes recovered for a pathway were mistakenly recruited to the genome bin (i.e., belong to the contaminant fraction). Given an estimate of contamination in a genome bin as assessed by CheckM, C, and the number of contigs containing genes in a pathway of interest recovered in the genome bin, k, the probability, P, that all of these genes do not actually belong to the genome is given by P = C^k^. In the genome bins recovered here, phototrophy genes are largely recovered on separate short contigs, and so k is typically equal to the number of phototrophy genes recovered. Following the example above, the likelihood that *pufL, pufM, pufC, bchX, bchY*, and *bchZ* were all mistakenly assigned to bin CP2_42A is *P* = 0.1042^6^ = 0.00000127998. This could also be considered a conservative estimate, as it ignores the fraction of contaminant genes that are due to strain-level heterogeneity rather than genes from unrelated organisms (16.13% in the case of CP2_42A).

## Results and discussion

Sequencing of both hot spring metagenomes and a cultured isolate yielded draft genomes of three new reaction center-containing phototrophic Chloroflexi lineages (*K. aurantiaca*, JP3_7, and CP2_42A). In addition to these new phototrophs, eight genome bins were recovered that are associated with a new class-level clade, sister to the Anaerolineae (Tables 1 – 3, Figure 1, Supplemental Figures 3, 4). *K. aurantiaca* represents a thus-far monospecific genus within the class Chloroflexia, basal to *Roseiflexus*; JP3_7 is a sister taxon to *Ca*. Roseilinea gracile; and CP2_42A and the other genome bins reported here form a new clade sister to the Anaerolineae. Genome statistics and summaries of the key metabolic proteins encoded by these genomes are reported in Tables 2, 3.

**Table 3:** Presence/absence of selected organismal markers and metabolic genes in genomes reported here.

	**16S**	**RpoB**	**Type 2 reaction center**	***bc* complex**	**ACIII**	**Rhodopsin**	**Denitrification**	**A-Family HCO**	**B-Family HCO**	**3HP**	**Calvin Cycle**
CP1_1M	–	+	–	+	–	–	–	–	–	–	–
CP2_2F	–	+	–	–	–	+	–	–	–	–	–
CP2_20G	–	+	–	+	–	–	nirK	+	–	–	–
CP2_42A	–	+	+ (fused)	+	+	+	–	+ (two)	+	–	–
JP1_8	–	+	–	–	–	–	–	–	–	–	–
JP1_16	–	+	–	+	–	–	–	+	–	–	–
JP1_20	–	+	–	+	–	–	nirK, NOR	+ (three)	–	–	–
JP1_191	–	+	–	-	–	–	–	–	–	–	–
JP3_7	–	+	+ (unfused)	+	–	–	–	+	+	–	–
JP3_13	–	+	–	+	–	+	–	+ (two)	–	–	–
*Kouleothrix aurantiaca*	+	+	+ (fused)	+	–	–	nirK	+	+	–	+

Organismal phylogenies of the Chloroflexi phylum, including the novel phototrophs and other draft genome described here, were constructed using conserved, single-copy protein sequences including RpoB (Figure 1, Supplemental Figure 4) and concatenated ribosomal proteins (Supplemental Figure 3). RpoB is a core information processing protein, found as a single copy, and offers a character set that is commonly vertically inherited (Hansmann and Martin, 2000), and moreover was recovered in even low-completion genome bins, allowing the placement of additional low completion genomes into *Ca*. Thermofonsia (Figure 1, Table 3). Concatenated ribosomal protein sequences provide a large, robust dataset for resolving organismal relationships, and were used following methods from Hug et al. (2016). Due to low genome completeness and the recovery of only a partial RpoB sequence, bin JP1_191 was not included in figures.

**Figure 2:**
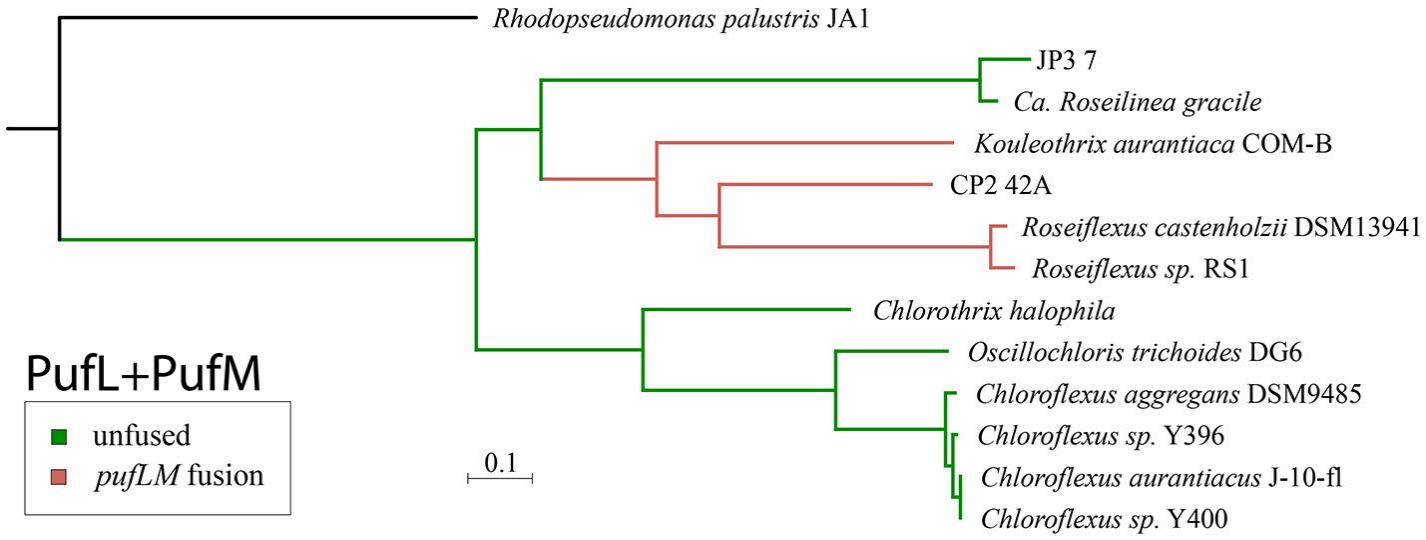
Phylogeny of Type 2 phototrophic reaction center proteins made from concatenated sequences of PufL and PufM. Lineages with a fused *pufLM* gene are highlighted in pink while lineages with unfused reaction center genes are shown in green. The phylogeny of reaction center proteins is incongruent with the organismal tree (Figure 1), suggesting a history of horizontal gene transfer. However, the monophyly of fused *pufLM* genes (pink) is consistent with a singular gene fusion event.

Phylogenetic trees of reaction center proteins (i.e., PufL and PufM) (Figure 2, Supplemental Figure 5) show *Kouleothrix* in the same position relative to other Chloroflexia as in organismal trees (i.e., basal to *Roseiflexus*), but these analyses place CP2_42A and JP3_7 very differently—with CP2_42A as branching between *Kouleothrix* and *Roseiflexus*, and JP3_7 branching sister to the *Roseiflexus*+CP2_42A+*Kouleothrix* clade.

### Kouleothrix aurantiaca

*K. aurantiaca* encodes for all of the genes required for anoxygenic phototrophy; a Type 2 reaction center (RC2) (including a fused *pufLM* and *pufC*), a complete bacteriochlorophyll biosynthesis pathway, and a cytochrome *bc* complex, but no Alternative Complex III. *K. aurantiaca* encodes a form 1 RuBisCO and a phosphoribulokinase gene, suggesting that it is capable of carbon fixation via the Calvin Cycle. It does not, however, encode key genes in the 3-hydroxypropionate bicycle (3HP) used for carbon fixation in *Chloroflexus* and *Roseiflexus* (Klatt et al., 2007; Shih et al., 2017). *K. aurantiaca* falls within the phototrophic Chloroflexia, with a consistent position basal to *Roseiflexus* in both organismal and photosynthetic gene trees (Figures 1, 2). This suggests that phototrophy is a synapomorphy of the Chloroflexales order (i.e., the members of the Chloroflexia class after the divergence of the basal members *Herpetosiphon* and *Kallotenue*), with a single acquisition at the base of the clade, before the divergence of the *Chloroflexus* and *Roseiflexus* lineages, with no known instances of loss of phototrophy.

### *Candidatus* thermofonsia—a metabolically diverse class of chloroflexi, sister to anaerolineae

Several of the draft genomes reported here (CP1_1M, CP2_2F, CP2_20G, CP2_42A, JP1_8, JP1_16, JP1_20, JP1_191, and JP3_13) cluster together in phylogenies based on organismal marker genes (e.g., RpoB, Figure 1, and concatenated ribosomal protein sequences, Supplemental Figure 3), forming a monophyletic clade sister to the Anaerolineae class. Additionally, JP3_7 and *Ca*. Roseilinea gracile (the “Anaerolineae-like” phototroph recovered from a Yellowstone National Park metagenome) (Klatt et al., 2011; Thiel et al., 2016, 2017; Tank et al., 2017) are tentatively assigned to this class as they cluster together under some analyses (e.g., RpoB, Figure 1), though in concatenated ribosomal protein phylogenies these strains cluster with *Thermoflexus hugenholtzii* in a lineage basal to Anaerolineae and the new class described here (Supplemental Figure 3). Genome analyses show that the members of the new class described here encode diverse heterotrophic metabolic traits, including photoheterotrophy and several pathways for both aerobic and anaerobic respiration (Table 3).

For this new clade, we propose the name *Candidatus* Thermofonsia, from the Latin for hot spring, and the suffix -*ia* for a class level, with official classification pending isolation and characterization of at least one member. The members of *Ca*. Thermofonsia described here fall into three lower-order clades in organismal trees, each composed of sequences from members recovered from multiple hot spring metagenomes. The monophyly of each of these clades was recovered in all organismal phylogenies even if relationships between them vary depending on analysis (e.g., placement of JP3_7 and *Ca* Roseilinea gracile, Figure 1 and Supplemental Figure 3). These clades appear to vary in their metabolic characteristics based on the genomes recovered so far, but are overall more similar to each other than they are to the neighboring Anaerolineae class. In particular, the Thermofonsia appear to have more abundant and diverse pathways for high potential metabolism, including aerobic respiration, reaction center-based photrophy, and denitrification.

### Shared characters of *Ca*. thermofonsia and divergence from anaerolineae

Based on analysis of fairly complete Thermofonsia genomes (>75% completeness, i.e., CP2_20G, CP2_42A, JP1_16, JP1_20, JP3_7, and JP3_13), several traits appear to be common characteristics of these organisms, likely inherited from the last common ancestor of the class (i.e., synapomorphies). Some of these traits (e.g., carotenoid synthesis) are shared with other Chloroflexi, while others (such as O_2_ metabolism) distinguish the Thermofonsia from their closest relatives.

The Thermofonsia described here possess many genes for metabolizing O_2_ that distinguish them from their sister class, Anaerolineae. The Anaerolineae are typically described as obligate anaerobes (e.g., Yamada and Sekiguchi, 2009), though genes for aerobic respiration have been recovered in a number of Anaerolineae genomes (e.g., Hemp et al., 2015a; Ward et al., 2015a). Phylogenetic analysis of electron transport and respiration genes in the Thermofonsia and Anaerolineae reveal metabolic protein trees that are incongruent with organismal relationships, implying independent acquisitions of respiration in these two clades (Supplemental Figures 6 – 9). The Thermofonsia identified thus far utilize a *bc* complex for respiration, while the Anaerolineae commonly use an Alternative Complex III (ACIII). Furthermore, the Heme Copper Oxidoreductases (HCOs) in these organisms are not closely related (Supplemental Figures 6, 7). Thermofonsia use a low-O_2_ affinity A-family HCO closely related to those of Cyanobacteria, while those in Anaerolineae are closely related to those found in the Chloroflexi class Caldilineae. The A-family HCOs found in Thermofonsia are closely related to each other, potentially reflecting vertical inheritance from their last common ancestor. These genes are not closely related to those of other members of the Chloroflexi, potentially reflecting acquisition of aerobic respiration at the base of the class, rather than at the origin of the phylum. This suggests that stem group lineages of these classes diverged prior to the acquisition of aerobic respiration, followed by diversification after receiving this metabolism through horizontal gene transfer, or alternatively loss and replacement (from a different source) in at least one lineage. Similarly, phylogenies of the *bc* complex in Thermofonsia (Supplemental Figure 8) largely recapitulate organismal relationships. In this case, however, the closest relatives of Thermofonsia sequences are those from other Chloroflexi, potentially reflecting an earlier acquisition of Complex III or intra-phylum HGT. Few Thermofonsia (only CP2_20G and JP3_13) encode *bd* oxidase enzymes used for respiration or O_2_ detoxification at low O_2_ concentrations (Borisov et al., 2011), whereas this enzyme is common in the Anaerolineae (Hemp et al., 2015c; Pace et al., 2015; Ward et al., 2015a). Moreover, the members of Thermofonsia reported here tend to encode fewer oxidative stress response genes than is typical for the Anaerolineae as annotated in RAST (mean of 10 vs. 19 among fairly complete Thermofonsia and Anaerolineae, respectively). Together these lines of evidence support interpretations of *Ca*. Thermofonsia being adapted to a more aerobic lifestyle than the Anaerolineae. It is therefore possible that the acquisition of aerobic respiration via HGT by early diverging ancestors of the Thermofonsia may have triggered diversification and radiation of this clade associated with invasion of newly accessible metabolic niches.

While some members of the Thermofonsia encode genes for nitrogen respiration (discussed below), other anaerobic respiration pathways—such as sulfate reduction—were not observed, nor were genes for bioenergetic nitrogen or sulfur oxidation. No Thermofonsia genomes described here contain genes for nitrogenase. Overall, the gene content of the Thermofonsia described here are characteristic with a lifestyle as aerobic heterotrophs.

Like other Chloroflexi, most members of *Ca*. Thermofonsia encode genes associated with carotenoid synthesis, such as phytoene synthase, phytoene desaturase, and lycopene cyclase, though these appear to be absent in JP1_16, despite the relative completeness of this genome, suggesting that carotenoid synthesis may be a common but not universal trait within this class. No genes for flagellar synthesis were identified in members of the Thermofonsia, but it is possible that they are capable of gliding motility like other members of the Chloroflexi. Marker genes for this trait have not yet been identified, but genes for chemotaxis regulation (e.g., CheA, CheR, CheY) are common.

### *Ca*. thermofonsia clade 1: JP3_13, CP1_1M, and CP2_2F

The first clade within *Ca*. Therofonsia is represented here by genome bins JP3_13, CP1_1M, and CP2_2F, of which JP3_13 is the most complete (~96% as estimated by CheckM). Members of this clade characterized thus far have GC content ~60% and predicted average estimated full genome size of ~3.7 Mb. While the CP1_1M and CP2_2F genomes are too incomplete for thorough metabolic characterization, JP3_13 was used here as representative of the clade. JP3_13 encodes a *bc* complex and two A-family Heme-Copper Oxidoreductases for aerobic respiration. CP2_20G and JP3_13 also contain genes for a *bd* oxidase, an O_2_ reductase adapted to low O_2_ concentrations. Two members of Clade 1 contain rhodopsin genes (CP2_2F and JP3_13). These rhodopsin genes have highly similar sequences, and were likely inherited from the last common ancestor of these strains. These rhodopsins are related to the “Actinorhodopsins” found in *Roseiflexus* sp. RS-1, which are thought to be functional as light-driven proton pumps (Sharma et al., 2008). Despite the presence of rhodopsins in diverse members of the Chloroflexi, including the Thermofonsia described here as well as *Roseiflexus, Ktedonobacter racemifer*, and *Bellilinea caldifistulae* (members of the Chloroflexia, Ktedonobacteraceae, and Anaerolineae classes of the Chloroflexi, respectively), the rhodopsins in each of these Chloroflexi lineages are not closely related, and likely reflect independent acquisitions via horizontal gene transfer from other phyla and not a shared history of rhodopsins in the Chloroflexi phylum.

### *Ca*. thermofonsia clade 2: CP2_20G, CP2_42A, JP1_8, JP1_16, JP1_20, and JP1_191

The second clade of Thermofonsia described here contains the genomes CP2_20G, JP1_20, CP2_42A, JP1_8, JP1_16, and JP1_191. Of these, JP1_16 is the most complete (~95%) and JP1_191 the least (~11%), while the others are of ~80% completeness. Due to its incompleteness and the recovery of only a partial RpoB sequence, JP1_191 was excluded from most figures and the following discussion. GC content of this clade appears to be typically lower than for Clade 1, ranging between 44 and 51% for most genomes with the single outlier of CP2_42A at 59%. The average predicted genome size (recovered genome divided by estimated completeness) is slightly larger than for Clade 1 (4.0 vs. 3.7 Mb).

This clade encompasses members with the potential for rhodopsin- and reaction center-based phototrophy (CP2_42A), partial denitrification (CP2_20G and JP1_20), and aerobic respiration (all genomes >50% completeness). Aerobic respiration in this clade is largely associated with A-family HCOs and *bc* complexes, consistent with Thermofonsia Clade 1 with whom these genes share a phylogenetic affinity (Supplemental Figures 6, 8). CP2_42A is the only member of this clade that contains genes for Alternative Complex III and a B-family HCO, potentially related to its capacity for phototrophy (see below). While CP2_42A appears to be capable of reaction center-based phototrophy (see below), no other members of this clade encode the necessary genes for phototrophy. JP1_8, the sister taxon to CP2_42A in organismal phylogenies, contains no marker genes for phototrophy; given the completeness of this genome, and the calculations for statistical confidence of the absence of genes from a metagenome bin, there is less than a 0.5% chance that this organism is a reaction center-based phototroph but the genes simply failed to be recovered in the genome bin (Supplemental Figure 12). While the presence of phototrophy in CP2_42A and absence in JP1_8 may be a result of presence in their last common ancestor followed by loss in JP1_8, it is equally parsimonious to assume HGT into CP2_42A from another source, a scenario that is discussed in greater detail below. CP2_42A encodes a rhodopsin gene most closely related to xanthorhodopsin, a proton-pumping rhodopsin shown to use light-harvesting antenna carotenoids (Balashov et al., 2005). CP2_42A also encodes a NiFe hydrogenase, a feature that was not recovered in any other Thermofonsia genomes.

### *Ca*. thermofonsia clade 3: JP3_7 and *Ca*. roseilinea gracile

The third clade of *Ca*. Thermofonsia consists of *Ca*. Roseilinea gracile, described elsewhere (e.g., Klatt et al., 2011; Tank et al., 2017), and JP3_7, described here. The phylogenetic placement of this clade is tentative, as it varies somewhat between marker sets (e.g., RpoB, Figure 1, and concatenated ribosomal proteins, Supplemental Figure 3). Both members of this clade described so far encode a Type 2 reaction center and genes for bacteriochlorophyll synthesis (discussed in detail below) but not genes for carbon fixation, suggesting a photoheterotrophic lifestyle. These organisms contain genes for aerobic respiration via both an A- and B-family HCO as well as a *bc* complex. Unlike other phototrophic Chloroflexi, Alternative Complex III was recovered not in these genomes.

### Anoxygenic phototrophy in chloroflexi outside the chloroflexia class

The draft genomes reported here include two organisms from outside the Chloroflexia class (JP3_7 and CP2_42A) that contain genes for anoxygenic phototrophy via Type 2 reaction centers (though some genes for bacteriochlorophyll synthesis were not recovered, see below).

JP3_7 is most closely related to *Ca*. Roseilinea gracile—the “Anaerolineae-like” phototrophic Chloroflexi assembled from a metagenome from Yellowstone National Park (Klatt et al., 2011; Thiel et al., 2016, 2017; Tank et al., 2017), though it is genetically distinct at the species and possibly the genus level (~78% average nucleotide identity across the genome, Goris et al., 2007). JP3_7 encodes genes for anoxygenic phototrophy: a Type 2 reaction center (including *pufL*, *pufM*, and *pufC*), bacteriochlorophyll *a* synthesis, and a cytochrome *bc* complex, but no Alternative Complex III. Interestingly, JP3_7 (as well as *Ca*. Roseilinea gracile) possesses fused *pufL* and *pufM* genes, a rare feature previously only observed in *Roseiflexus* (Youvan et al., 1984; Yamada et al., 2005). JP3_7 and *Ca*. Roseilinea gracile branch with *Ca*. Thermofonsia in RpoB phylogenies (Figure 1), albeit with weak bootstrap support (Supplemental Figure 4). However, in concatenated ribosomal protein trees, JP3_7 and *Ca*. Roseilinea gracile cluster with *T. hugenholtzii* as a lineage branching basal to Anaeolineae+Thermofonsia (Supplemental Figure 3). Note that the uncertainty in the exact placement of this lineage does not affect interpretations of evolutionary relationships of these organisms (e.g., HGT of phototrophy genes, see below).

CP2_42A encodes genes for anoxygenic phototrophy; a Type 2 reaction center (including a fused *pufLM* and *pufC*), bacteriochlorophyll *a* biosynthesis, a cytochrome *bc* complex, and Alternative Complex III. CP2_42A falls within *Ca*. Thermofonsia, and is separated from its closest phototrophic relatives (JP3_7 and *Ca*. Roseilinea gracile) by several nonphototrophic lineages (Figure 1).

While the draft genomes reported here are largely too fragmented to recover informational genes on the same contigs as phototrophy related genes, the *rpoB* and *bchP* genes of JP3_7 were collocated on the same contig, providing strong support for the inference of phototrophy in this lineage from these genome bins. Moreover, given the relatively low contamination in these genome bins as estimated by CheckM (<13% in both JP3_7 and CP2_42A, much of which is due to strain-level heterogeneity rather than contamination from unrelated organisms, Table 2), the likelihood of multiple contigs bearing phototrophy-related genes being mistakenly assigned to these genome bins is low (e.g., 10^−6^ for *pufL, pufM, pufC, bchX, bchY*, and *bchZ* in CP2_42A), providing statistical confidence that phototrophy genes belong to these genome bins.

While other phototrophic Chloroflexi encode carbon fixation via the 3-hydroxypropionate pathway (e.g., *Chloroflexus)* or the Calvin Cycle (e.g., *Oscillochloris*) (Shih et al., 2017), these pathways are absent from the draft genomes of CP2_42A, JP3_7, and *Ca*. Roseilinea gracile, potentially reflecting a lifestyle as photoheterotrophs.

### Horizontal gene transfer of phototrophy within the chloroflexi

The position of *Kouleothrix* in both organismal and gene trees is consistent with a vertical inheritance of phototrophy from the last common ancestor of the *Roseiflexus*+*Chloroflexus* clade after its divergence from the nonphototrophic *Herpetosiphon* and *Kallotenue*. However, the other two phototrophic Chloroflexi reported here, along with *Ca*. Roseilinea gracile reported previously, reveal a more complex history. In organismal trees based on conserved vertically inherited proteins (e.g., RpoB, Figure 1, or concatenated ribosomal proteins, Supplemental Figure 3), these two strains (CP2_42A and JP3_7) sit well outside the Chloroflexia class where other phototrophic Chloroflexi are found, separated by many nonphototrophic lineages (Figure 1). However, phylogenetic relationships of phototrophy-related genes (such as reaction centers and bacteriochlorophyll synthesis genes) place these strains within clades comprised of other phototrophic Chloroflexi (Figure 2, Supplemental Figures 10, 11). In reaction center protein trees, CP2_42A branches within the Chloroflexia, basal to the clade of *Roseiflexus* and *Kouleothrix*. JP3_7, however, branches more deeply, sister to the *Roseiflexus*+*Kouleothrix*+CP2_42A clade (Figure 2). Furthermore, *Kouleothrix* and CP2_42A have fused *pufL* and *pufM* genes, a feature which appears in reaction centers of *Roseiflexus* (Youvan et al., 1984; Yamada et al., 2005), and so appears to be a synapomorphy of this lineage of phototrophs, supporting their inclusion at this point in the phototrophy tree to the exclusion of JP3_7 (which has unfused *pufL* and *pufM* genes). This also suggests that the *pufLM* fusion is a rare event, and therefore the presence of the fused form of these genes is a useful trait for assessing the relatedness of reaction centers independent of overall sequence similarity.

The discordance of the topologies between organismal (e.g., concatenated ribosomal protein) and phototrophy (e.g., reaction center protein) trees for the novel phototrophic Chloroflexi described here suggests that phototrophy genes were not vertically inherited from the last common ancestor of the phototrophic Chloroflexi. Instead, the differing branching order of JP3_7 and CP2_42A between organismal (e.g., concatenated ribosomal protein) and phototrophy (e.g., reaction center protein) trees, along with the presence of a conserved gene fusion within the *Roseiflexus*+*Kouleothrix*+CP2_42A clade, strongly suggests that horizontal gene transfer has played a role in the current distribution of phototrophy in the Chloroflexi phylum.

**Figure 3:**
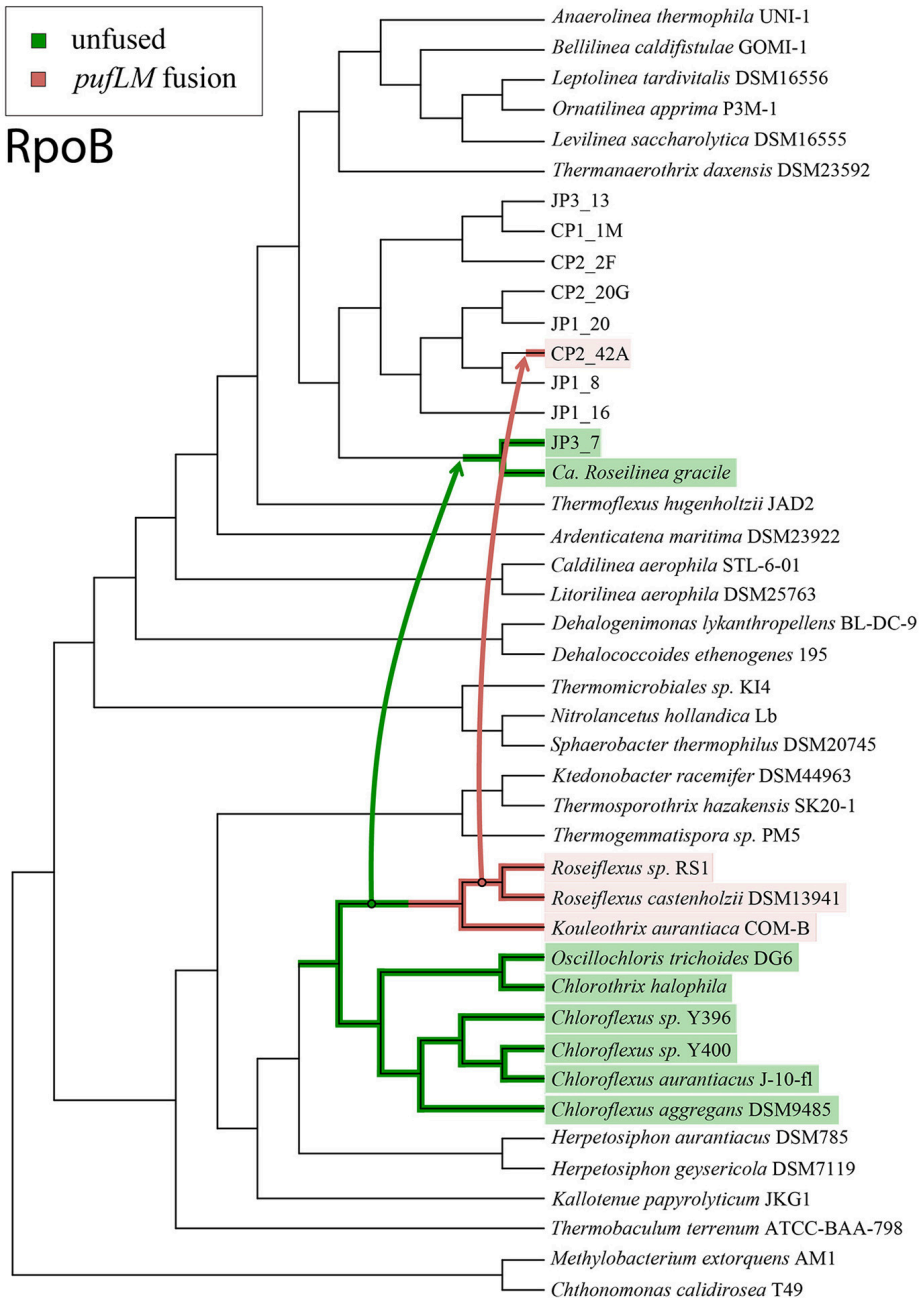
Cladogram of the Chloroflexi phylum based on RpoB protein sequences, illustrated with the simplest possible evolutionary history of phototrophy that honors the relationships between the reaction center proteins and organismal markers. Non-phototrophic lineages are shown in black, lineages with fused *pufLM* reaction center genes are highlighted in pink, and lineages with unfused reaction center genes are shown in green. Arrows mark the inferred horizontal gene transfers of phototrophy genes. The most parsimonious scenario for the evolution of phototrophy within the Chloroflexi requires two separate horizontal gene transfer events, and a single gene fusion of *pufLM*.

In light of these data, the simplest scenario for the evolution of phototrophy within the Chloroflexi requires at least two instances of horizontal gene transfer to have occurred (Figure 3). In this scenario, the acquisition of an unfused Type 2 reaction center (and other phototrophy-related genes, such as those for bacteriochlorophyll synthesis) occurred in an ancestor of the phototrophic Chloroflexia after their divergence from *Herpetosiphon* and *Kallotenue*. Then, horizontal gene transfer of phototrophy, including an unfused ancestral Type 2 reaction center, from the branch leading to *Roseiflexus* into the JP3_7 lineage occurred, followed by a single *pufL*+*pufM* fusion event in the lineage leading to *Roseiflexus* and *Kouleothrix*. Finally, there was a second horizontal gene transfer event of phototrophy, including the now fused *pufLM* reaction center gene, into an ancestor of CP2_42A from the *Roseiflexus* lineage; phylogenetic relationships of the reaction centers show that this must have occurred after their divergence from *Kouleothrix*. It is important to note that this is the most parsimonious interpretation that honors all of the phylogenetic data. More complex scenarios involving more than two instances of HGT, or extensive HGT in addition to multiple losses, can also be envisioned.

### Presence and HGT of other physiologically relevant genes

The genome bins for CP2_42A and JP3_7 recover most, but not all, of the bacteriochlorophyll synthesis pathway expected for phototrophic Chloroflexi. These genomes contain *bchX, bchY, bchZ, bchP, bchF, bchG, bchI, bchD*, and a *bchH*-like gene, but not *bchL, bchN, bchB, bchM*, or *bchE*. While this may be a result of the incompleteness of these genomes, the same bacteriochlorophyll synthesis gene complement has been described in *Ca*. Roseilinea gracile (Klatt et al., 2011). Microscopic analysis of organisms tentatively identified as *Ca*. Roseilinea gracile has confirmed that it exhibits fluorescence characteristic of bacteriochlorophyll *a* (but not bacteriochlorophyll *c*), consistent with predictions based on genome content (Tank et al., 2017), suggesting that this organism is capable of bacteriochlorophyll synthesis despite its reduced gene complement. It is possible that some or all of these genes may actually be absent from these genomes, functionally replaced by promiscuous homologs (e.g., *bchL, bchN*, and *bchB* are homologous to *bchX, bchY*, and *bchZ*, and chimeras of other homologs of these genes have been demonstrated to be functionally exchangeable, e.g., Cheng et al., 2005; Wätzlich et al., 2009). While *bchE* can be functionally replaced by *acsF*, and *bchL, bchN*, and *bchB* can be functionally replaced by the light-dependent POR enzyme (Chew and Bryant, 2007), these genes were also not recovered in *Ca*. Roseilinea gracile, JP3_7, or CP2_42A genomes. Our estimates of the probabilities of missing the same set of genes from multiple genomes of relatively high (>50%) completeness are incredibly low (<<1%); this supports the hypothesis that these genes truly are absent from JP3_7, CP2_42A, and *Ca*. Roseilinea gracile (Supplemental Figure 12). Ultimately, isolation and biochemical characterization of the bacteriochlorophyll synthesis pathway in these organisms will be necessary to test this notion.

Phylogenies of electron transport proteins reveal that aerobic respiration using an A-family HCO (Supplemental Figure 6) and a *bc* complex was acquired at the base of the *Ca*. Thermofonsia class and has since been a vertically-inherited synapomorphy (Supplemental Figure 8), while the B-family HCO (Supplemental Figure 7) and Alternative Complex III (Supplemental Figure 9) found in phototrophic strains appear to have been acquired later through horizontal gene transfer associated with the acquisition of Type 2 reaction centers. These trends are consistent with those previously observed in the Chloroflexia class (Shih et al., 2017), and suggest that HGT and acquisition of metabolic traits such as respiration may be responsible for driving class-level radiations in the Chloroflexi phylum.

Interestingly, genes involved in lipopolysaccharide synthesis (e.g., *lpxB, lpxC, kdsA*) and outer membrane proteins (e.g., *bamA*) were absent from all Chloroflexi genomes reported here. This is consistent with the proposed single membrane “monoderm” nature of Chloroflexi (Sutcliffe, 2010, 2011) and supports the hypothesis that this is indeed a conserved feature of the Chloroflexi phylum, though the presence of outer membrane proteins and lipopolysaccharide synthesis in the closely related Armatimonadetes phylum (e.g., Ward et al., 2017) also implies that monoderm Chloroflexi may be derived from a diderm ancestor and are not representative of broader ancestral state of the superphylum.

It is also notable that the vast majority of sequenced phototrophic Chloroflexi utilize Alternative Complex III (Yanyushin et al., 2005) for energy conservation during phototrophic electron transport—even to the extent of CP2_42A having acquired ACIII along with phototrophy genes. On the other hand, ACIII was not recovered in the draft genomes for *K. aurantiaca* or JP3_7. This suggests that the use of ACIII for phototrophy may not be universal among phototrophic Chloroflexi, though this will require closure of these genomes and confirmation that ACIII is truly absent and not simply missing from the draft genome. The presence of auracyanin, the typical electron acceptor of ACIII (Majumder et al., 2013), in JP3_7 is consistent with the ancestral presence of ACIII in this lineage and either recent loss or failure to recover the gene in the genome bin. Meanwhile, all of the aerobic members of *Ca*. Thermofonsia encode a *bc* complex, consistent with other aerobic, nonphototrophic Chloroflexi clades such as Caldilineae and Ardenticatenia (e.g., Hemp et al., 2015b).

The history of carbon fixation in the Chloroflexi is also complex. While phototrophic Chloroflexi such as *Chloroflexus* and *Roseiflexus* are well known to possess the 3-hydroxypropionate bicycle for carbon fixation (e.g., Berg, 2011), this pathway is absent in the genomes reported here, as well as *Oscillochloris* and *Chlorothrix*. Instead, *Kouleothrix, Oscillochloris*, and *Chlorothrix* possess the Calvin Cycle (as indicated by the presence of RuBisCO and phosphoribulokinase genes), while CP2_42A and JP3_7 (along with *Ca*. Roseilinea gracile) do not appear to encode any carbon fixation pathways (suggesting a photoheterotrophic lifestyle). Overall, phototrophy and carbon fixation in the Chloroflexi appear to have largely independent histories, though both are largely driven by HGT (Shih et al., 2017).

## Conclusions

Here, we have added to the rapidly expanding genetic diversity of the Chloroflexi phylum with description of *Ca*. Thermofonsia, a new class-level clade. This class contains members with diverse high-potential metabolic pathways including aerobic respiration, denitrification, and phototrophy, distinguishing it from its sister class Anaerolineae. We have previously sequenced the genomes of diverse representatives of the Chloroflexi, filling in gaps in the tree (Hemp et al., 2015a,b,c; Pace et al., 2015; Ward et al., 2015a,b) in order to better characterize the diversity, distribution, and evolution of high potential metabolism within this phylum. These datasets have revealed a high degree of previously unrecognized metabolic diversity in this phylum, including high-potential metabolic pathways for aerobic and anaerobic respiration (Hemp et al., 2015a,b,c; Ward et al., 2015a,b). It is becoming apparent that the same is true for phototrophy. Together, these data are consistent with a high degree of metabolic diversity in Chloroflexi—driven in part by horizontal gene transfer of metabolic genes, including those for carbon fixation (Shih et al., 2017) and key components of high potential electron transport chains as described here.

The distribution of phototrophy within the Chloroflexi via HGT is similar to that observed in the Proteobacteria, which records extensive intra-phylum HGT (Igarashi et al., 2001; Swingley et al., 2009; Nagashima and Nagashima, 2013). A single clear case of inter-phylum HGT is also recorded in the presence of a Proteobacteria-derived Type 2 reaction center in a member of the Gemmatimonadetes (Zeng et al., 2014). It therefore appears that HGT has played a significant role in determining the modern distribution of anoxygenic phototrophy across the bacterial tree—consistent with the hypothesis of Raymond et al. (2002).

From comparative biochemistry and structural biology it is clear that some form of anoxygenic phototrophy preceded oxygenic photosynthesis (e.g., Hohmann-Marriott and Blankenship, 2011; Fischer et al., 2016), and thus at least one lineage must have acquired anoxygenic phototrophy before the Great Oxygenation Event (GOE) ~2.3 billion-years-ago. However, it is not clear which—if any—of the extant taxa with phototrophic members would have diverged and been present prior to the GOE. For many phototrophic groups, it was hypothesized that the acquisition of phototrophy postdated the acquisition of aerobic respiration—a mechanic enabled by the modular nature of high potential electron transport chains and shared machinery between aerobic respiration and phototrophy (Fischer et al., 2016). Our data is consistent with an initial acquisition of phototrophy in Chloroflexi lineages already capable of aerobic respiration; if this is correct, it suggests that phototrophy in this phylum must postdate the evolution of oxygenic photosynthesis and subsequent origin of aerobic respiration (e.g., Soo et al., 2017). As a result, Chloroflexi are unlikely to have been the inventors of anoxygenic phototrophy (Oyaizu et al., 1987), but instead acquired phototrophy via HGT, likely sometime after the GOE—perhaps as recently as ~1 Ga (Shih et al., 2017). It has been suggested that similar patterns will hold for other extant groups of anoxygenic phototrophs (Fischer et al., 2016). The taxonomic affinity of anoxygenic phototrophs on the early Earth, before the GOE, remains uncertain. It is possible that phototrophy originated in a thus-far undiscovered but still extant group, but it is also valuable to seriously consider the possibility that phototrophy first evolved in a now extinct stem lineage. These different hypotheses can best be resolved by continued discovery of new phototrophic groups—an increasingly frequent phenomenon as environmental sequencing efforts continue and improve.

## Data availability

Genomes described here have been deposited at GenBank and are available under the following accession numbers: *K. aurantiaca* (LJCR00000000), CP1_1M (PGTL00000000), CP2_20G (PGTJ00000000), CP2_2F (PGTK00000000), CP2_42A (PGTI00000000), JP1_16 (PGTG00000000), JP1_191 (PGTE00000000), JP1_20 (PGTF00000000), JP1_8 (PGTH00000000), JP3_13 (PGTM00000000), and JP3_7 (PGTN00000000).

## Author contributions

LW, SM, and WF: conceived the initial study; LW and SM: collected samples; LW: processed samples and data; LW, JH, PS, SM, and WF: analyzed data; LW, JH, PS, SM, and WF: wrote the manuscript.

### Conflict of interest statement

The authors declare that the research was conducted in the absence of any commercial or financial relationships that could be construed as a potential conflict of interest.

## Supplementary material

Supplemental Figure 1 Photos of Jinata Onsen. **(A)** Panorama of field site, with source pool on left (Pool 1 below), Pool 2 and 3 in center, and Out Flow to bay on right. **(B)** Undistorted view north up the canyon. **(C)** Undistorted view south toward bay, overlooking Pool 2. **(D)** Source pool, coated in floc-y iron oxides and bubbling with gas mixture containing H_2_, CO_2_, and CH_4_. **(E)** Pool 2, with mixture of red iron oxides and green from Cyanobacteria-rich microbial mats. **(F)** Close up of textured microbial mats in Pool 3. **(G)** Close up of Out Flow, where hot spring water mixes with ocean water.

Supplemental Figure 2 Photos of cone-forming microbial mats at Nakabusa Onsen, source of CP genome bins. **Top**: CP1, a cone-forming microbial mat growing at 48°C, whose fabric was made up of filamentous Chloroflexi. **Bottom**: CP2, a cone-forming microbial mat growing at 32°C, whose fabric was made up of filamentous Cyanobacteria.

Supplemental Figure 3 Concatenated ribosomal protein tree with bootstrap values, following methods from Hug et al. (2016).

Supplemental Figure 4 RpoB phylogeny from Figure 1 with bootstrap values.

Supplemental Figure 5 PufLM phylogeny from Figure 2 with bootstrap values.

Supplemental Figure 6 Unrooted phylogeny of A-family Heme Copper Oxidoreductase protein sequences. Sequences from *Ca*. Thermofonsia form a clade related to that from *Gloeobacter violaceus*, with the exception of JP3_7. This is consistent with a single acquisition of aerobic respiration near the base of Thermofonsia, followed by vertical inheritance into most strains.

Supplemental Figure 7 Unrooted phylogeny of B-family Heme Copper Oxidoreductase protein sequences. All phototrophic Chloroflexi encode B family HCOs, though the sequences do not appear to be closely related, likely reflecting independent histories and acquisition via HGT from different sources.

Supplemental Figure 8 Unrooted phylogeny of *bc* complex protein sequences. The phylogeny of *bc* complexes in Thermofonsia are largely congruent with those of A family HCOs, suggesting that these have a shared evolutionary history.

Supplemental Figure 9 Unrooted phylogeny of Alternative Complex III protein sequences. Most phototrophic Chloroflexi encode Alternative Complex III, though the sequences do not appear to be closely related, likely reflecting independent histories and acquisition via HGT from different sources.

Supplemental Figure 10 Unrooted phylogeny of concatenated BchXYZ protein sequences.

Supplemental Figure 11 Unrooted phylogeny of concatenated BchIDH protein sequences.

Supplemental Figure 12 “False negative” rate, or probability of failure to recover phototrophy genes, for a given completeness of genome recovery. Results plotted here are for a simulation following the constraints and logic discussed in the text.

Supplemental Table 1 Metagenome statistics.
