# Supplementary figures and images for: Evolution of Phototrophy in the Chloroflexi Phylum Driven by Horizontal Gene Transfer
Source: Front Microbiol. 2018 Feb 19;9:260. doi: 10.3389/fmicb.2018.00260 (PMC5826079; doi:10.3389/fmicb.2018.00260)

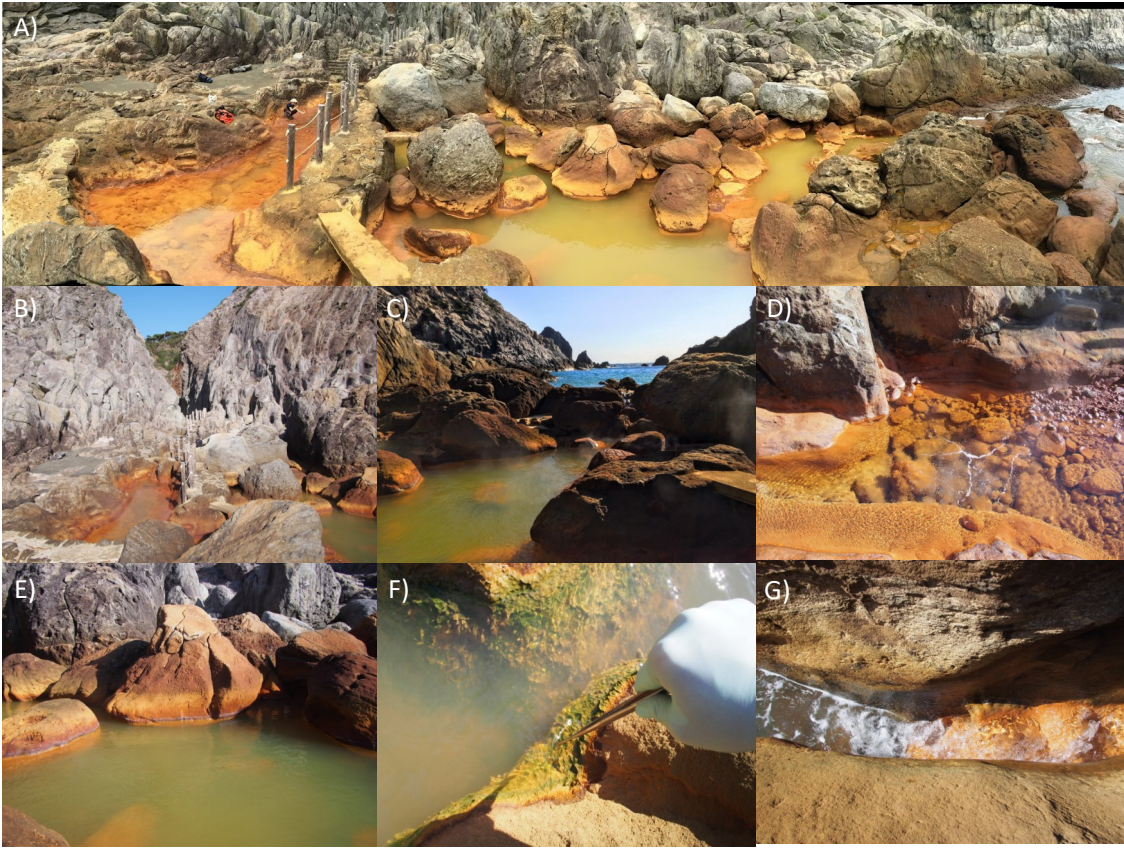

Supplement: Supplemental Figure 1 — Photos of Jinata Onsen. (A) Panorama of field site, with source pool on left (Pool 1 below), Pool 2 and 3 in center, and Out Flow to bay on right. (B) Undistorted view north up the canyon. (C) Undistorted view south toward bay, overlooking Pool 2. (D) Source pool, coated in floc-y iron oxides and bubbling with gas mixture containing H2, CO2, and CH4. (E) Pool 2, with mixture of red iron oxides and green from Cyanobacteria-rich microbial mats. (F) Close up of textured microbial mats in Pool 3. (G) Close up of Out Flow, where hot spring water mixes with ocean water. [file Image1.PDF]

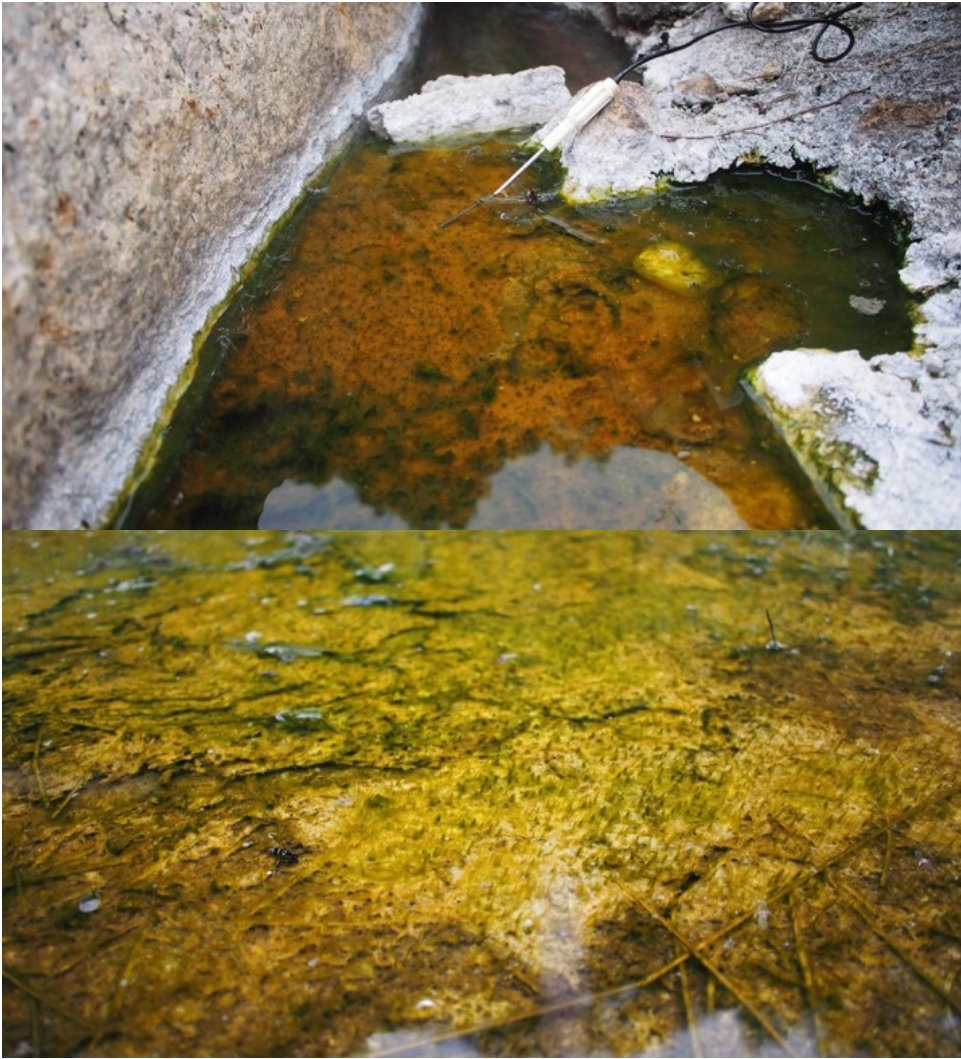

Supplement: Supplemental Figure 2 — Photos of cone-forming microbial mats at Nakabusa Onsen, source of CP genome bins. Top: CP1, a cone-forming microbial mat growing at 48°C, whose fabric was made up of filamentous Chloroflexi. Bottom: CP2, a cone-forming microbial mat growing at 32°C, whose fabric was made up of filamentous Cyanobacteria. [file Image2.PDF]

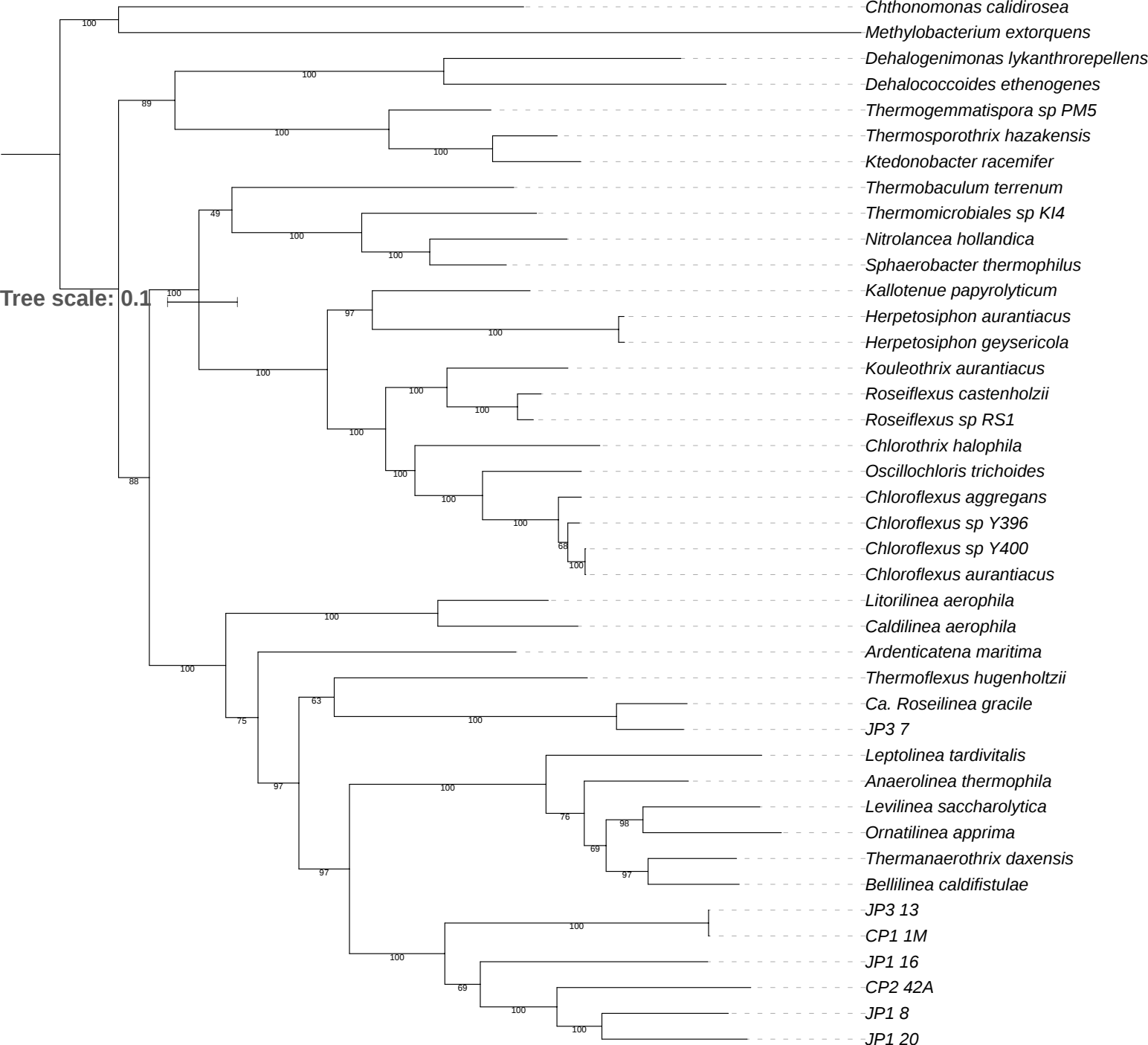

Supplement: Supplemental Figure 3 — Concatenated ribosomal protein tree with bootstrap values, following methods from Hug et al. (2016). [file Image3.PDF]

Tree scale: 0.1

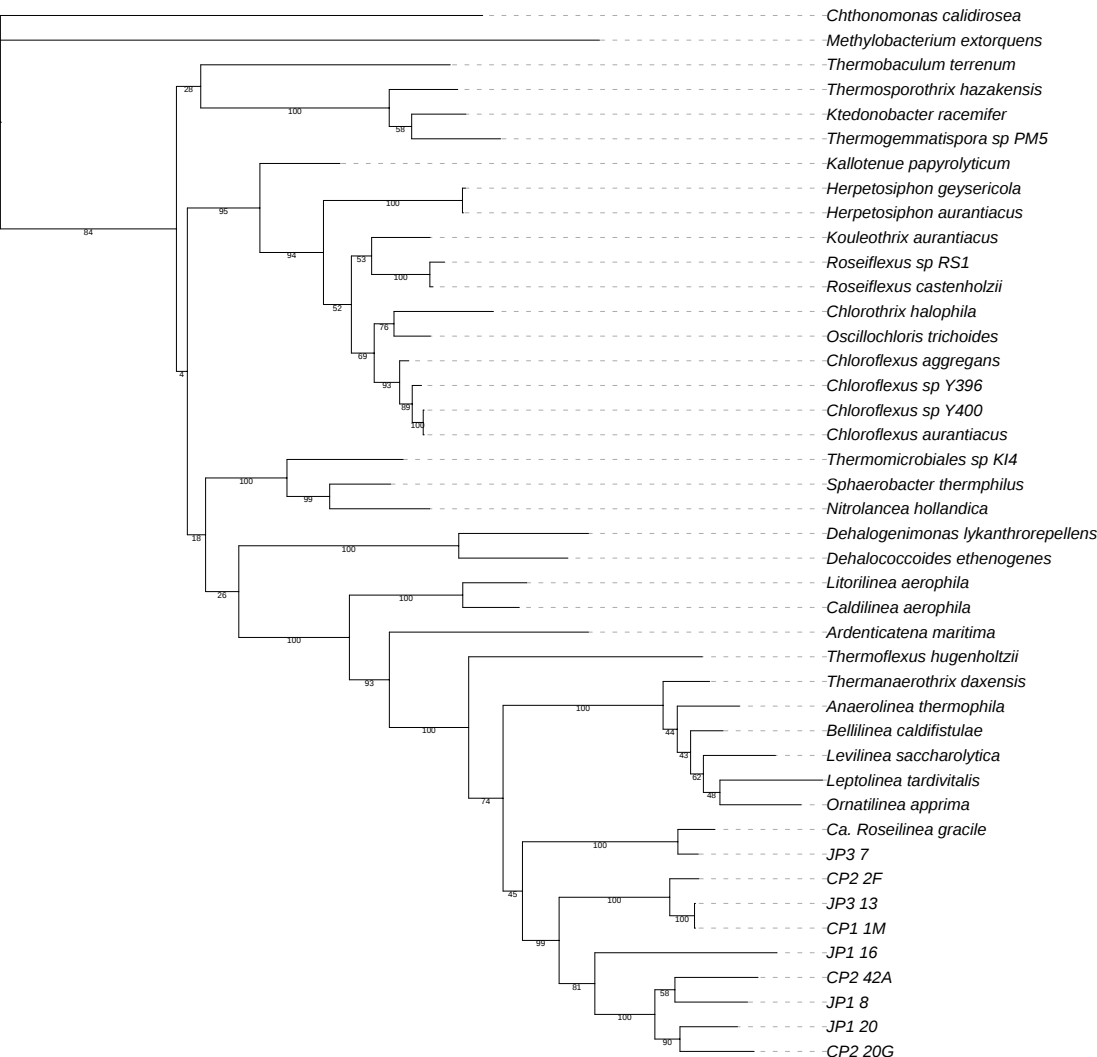

Supplement: Supplemental Figure 4 — RpoB phylogeny from Figure 1 with bootstrap values. [file Image4.PDF]

Tree scale: 0.1

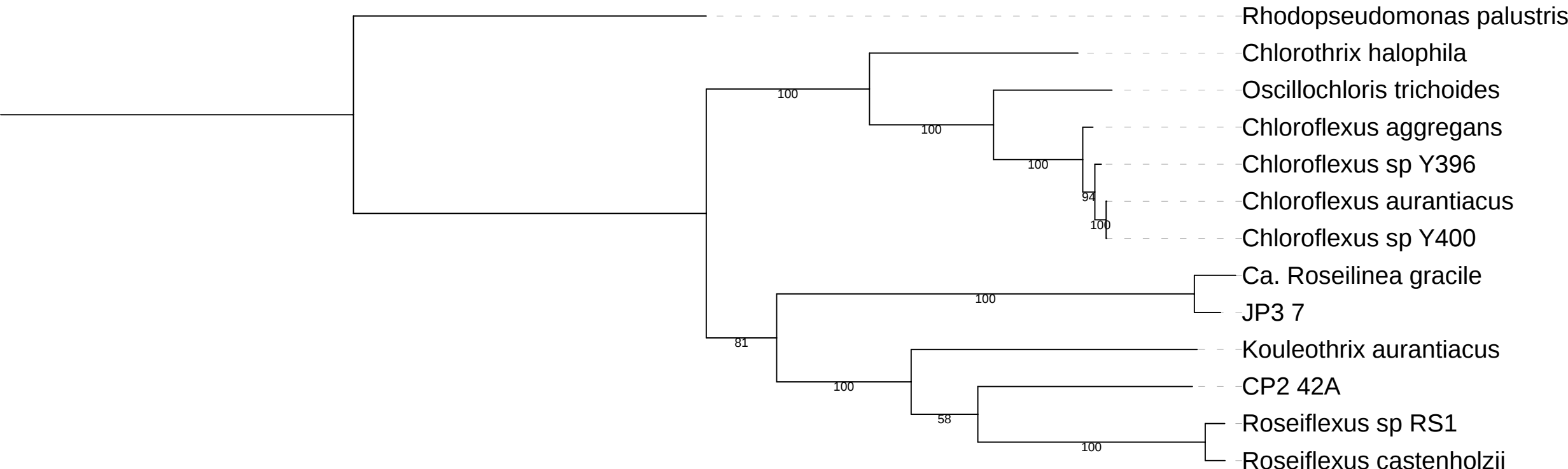

Supplement: Supplemental Figure 5 — PufLM phylogeny from Figure 2 with bootstrap values. [file Image5.PDF]

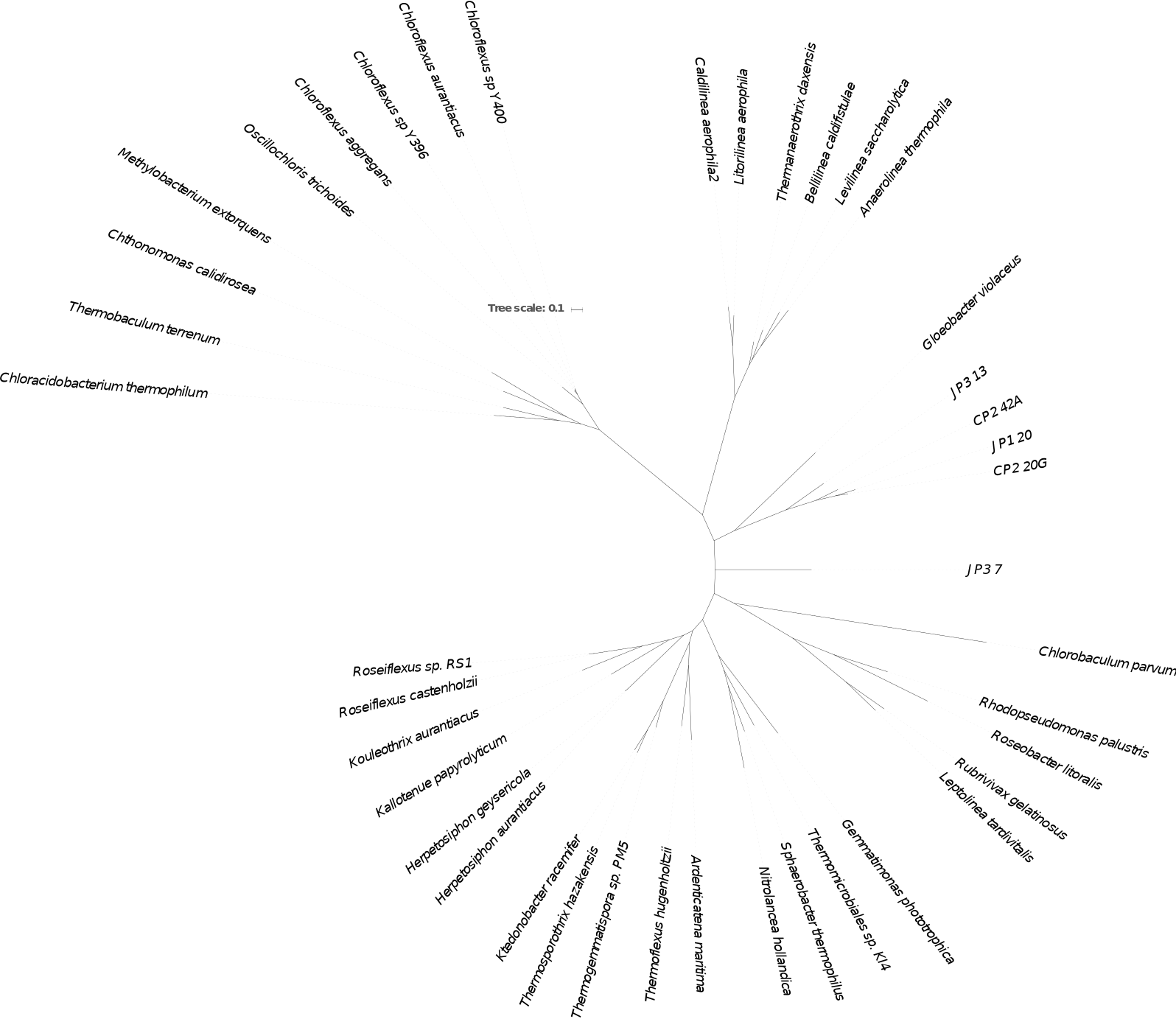

Supplement: Supplemental Figure 6 — Unrooted phylogeny of A-family Heme Copper Oxidoreductase protein sequences. Sequences from Ca. Thermofonsia form a clade related to that from Gloeobacter violaceus, with the exception of JP3_7. This is consistent with a single acquisition of aerobic respiration near the base of Thermofonsia, followed by vertical inheritance into most strains. [file Image6.PNG]

Tree scale: 1

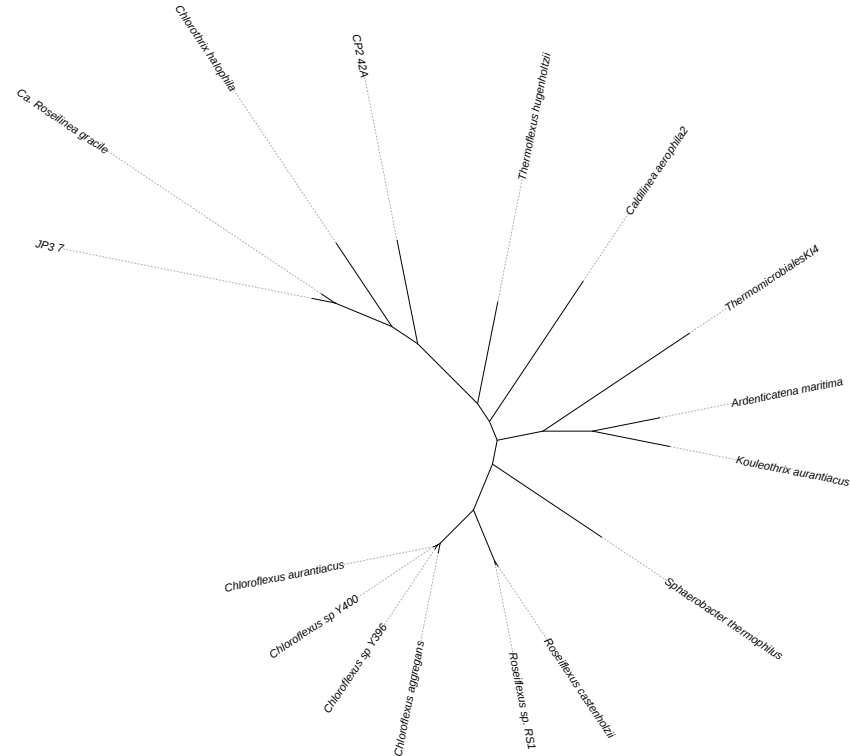

Supplement: Supplemental Figure 7 — Unrooted phylogeny of B-family Heme Copper Oxidoreductase protein sequences. All phototrophic Chloroflexi encode B family HCOs, though the sequences do not appear to be closely related, likely reflecting independent histories and acquisition via HGT from different sources. [file Image7.PDF]

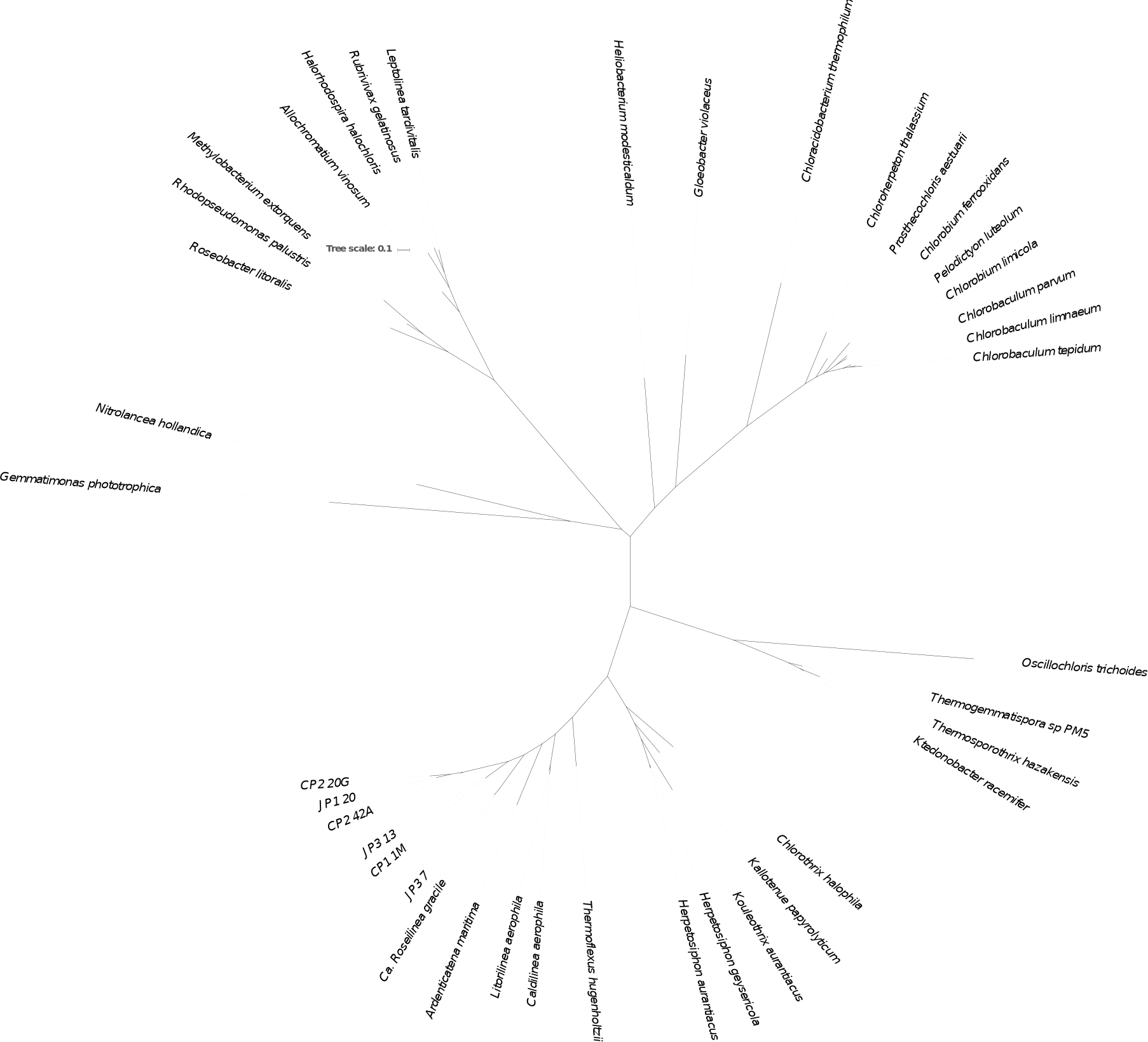

Supplement: Supplemental Figure 8 — Unrooted phylogeny of bc complex protein sequences. The phylogeny of bc complexes in Thermofonsia are largely congruent with those of A family HCOs, suggesting that these have a shared evolutionary history. [file Image8.PNG]

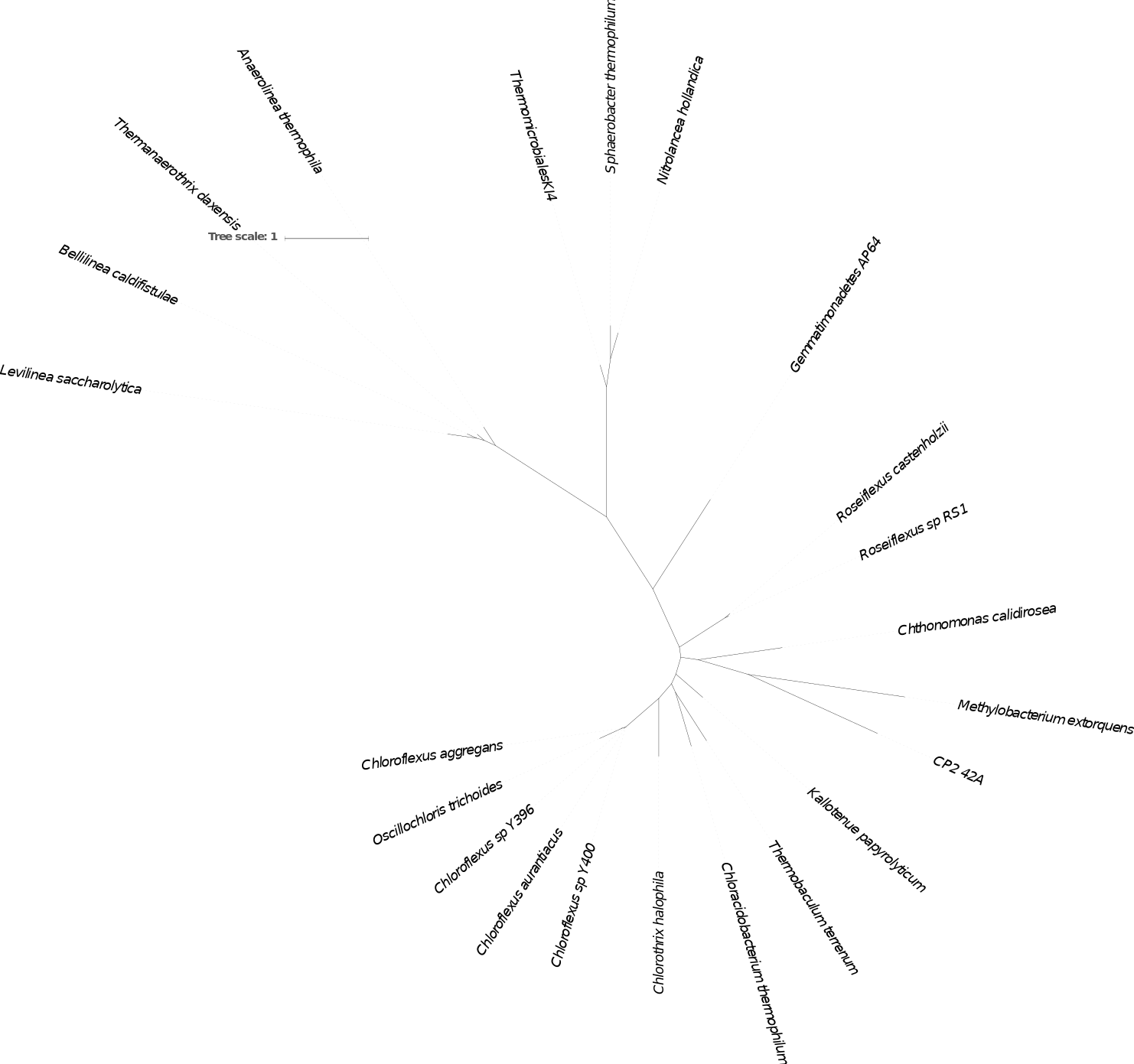

Supplement: Supplemental Figure 9 — Unrooted phylogeny of Alternative Complex III protein sequences. Most phototrophic Chloroflexi encode Alternative Complex III, though the sequences do not appear to be closely related, likely reflecting independent histories and acquisition via HGT from different sources. [file Image9.PNG]

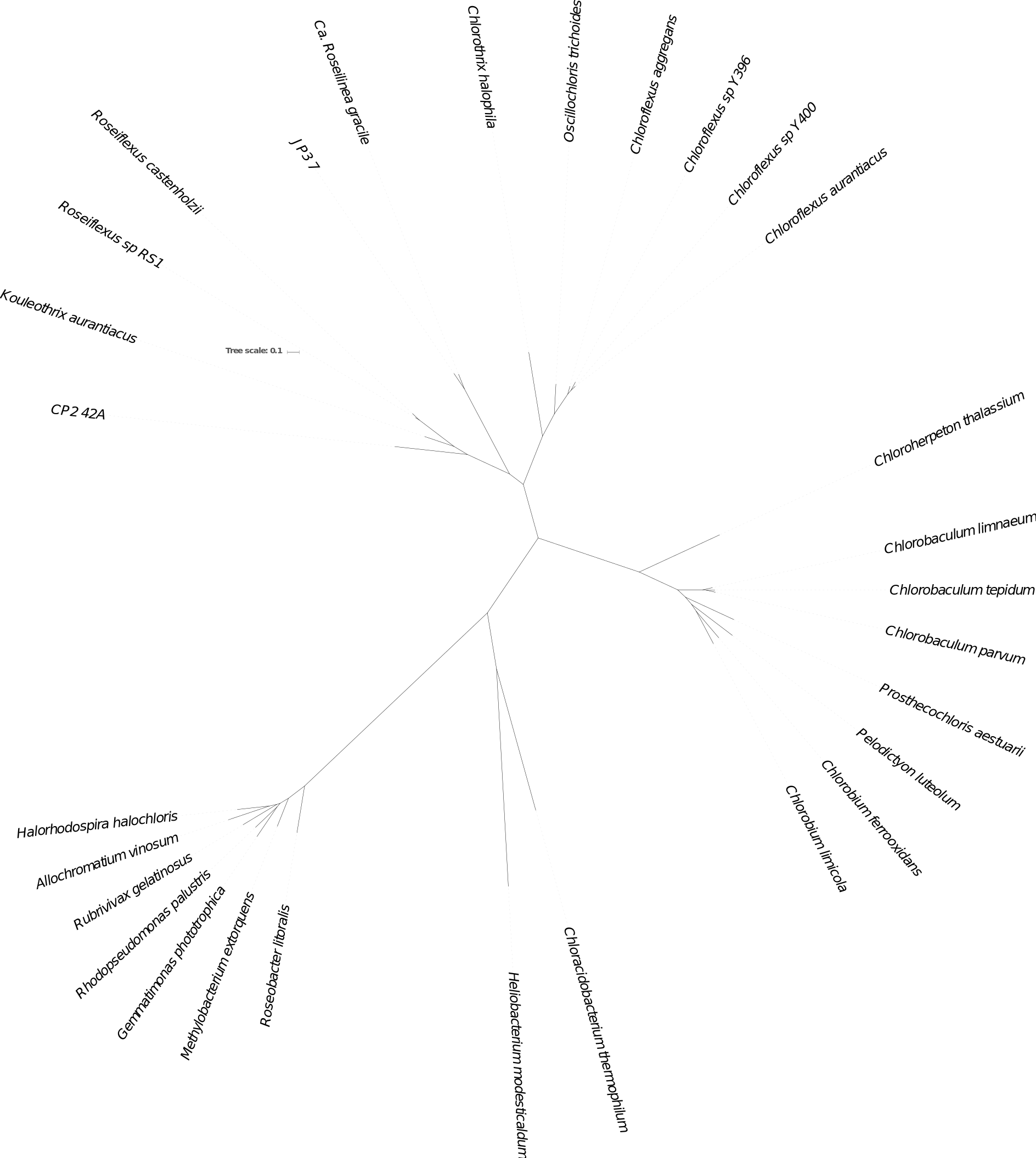

Supplement: Supplemental Figure 10 — Unrooted phylogeny of concatenated BchXYZ protein sequences. [file Image10.PNG]

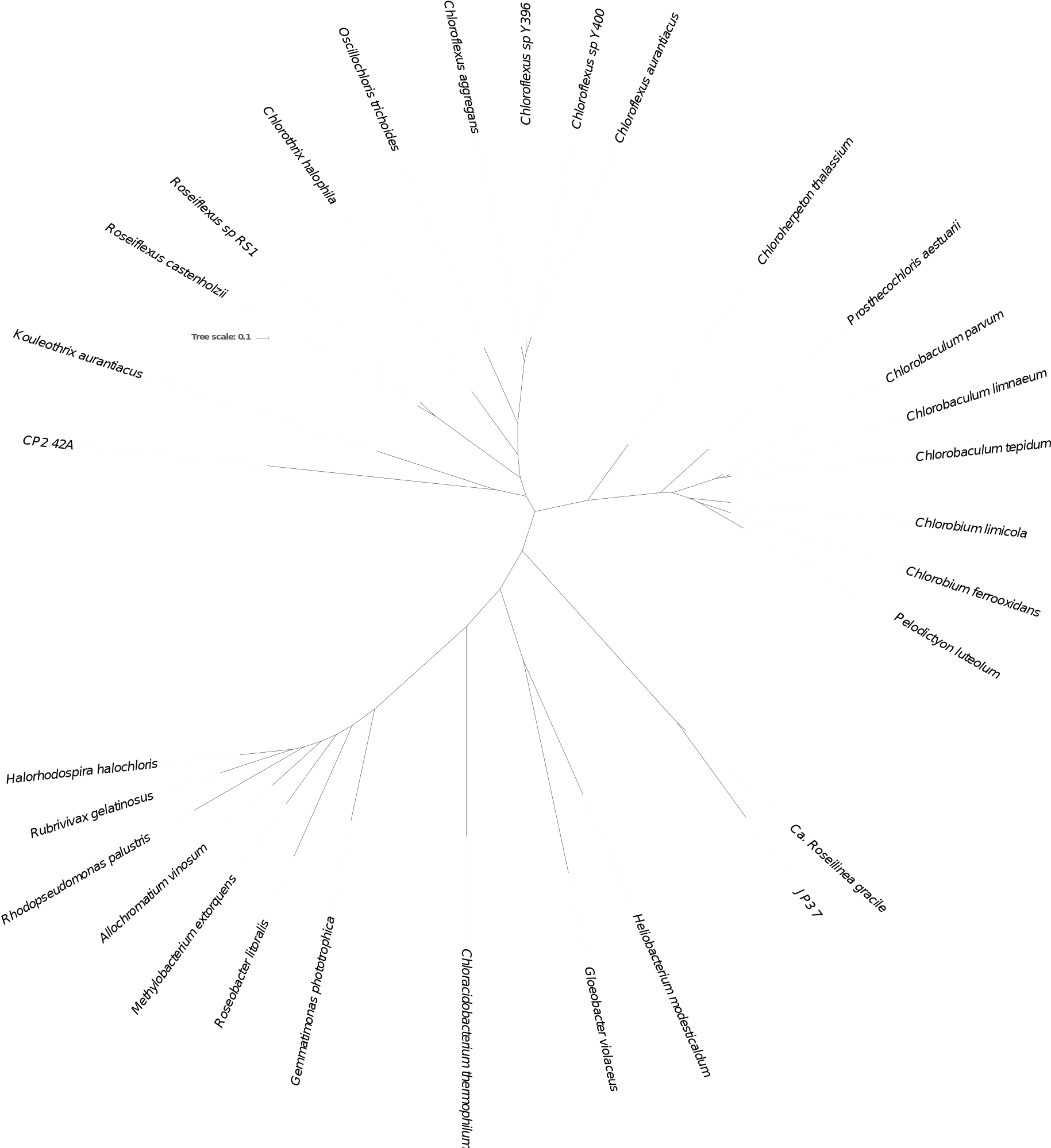

Supplement: Supplemental Figure 11 — Unrooted phylogeny of concatenated BchIDH protein sequences. [file Image11.PNG]

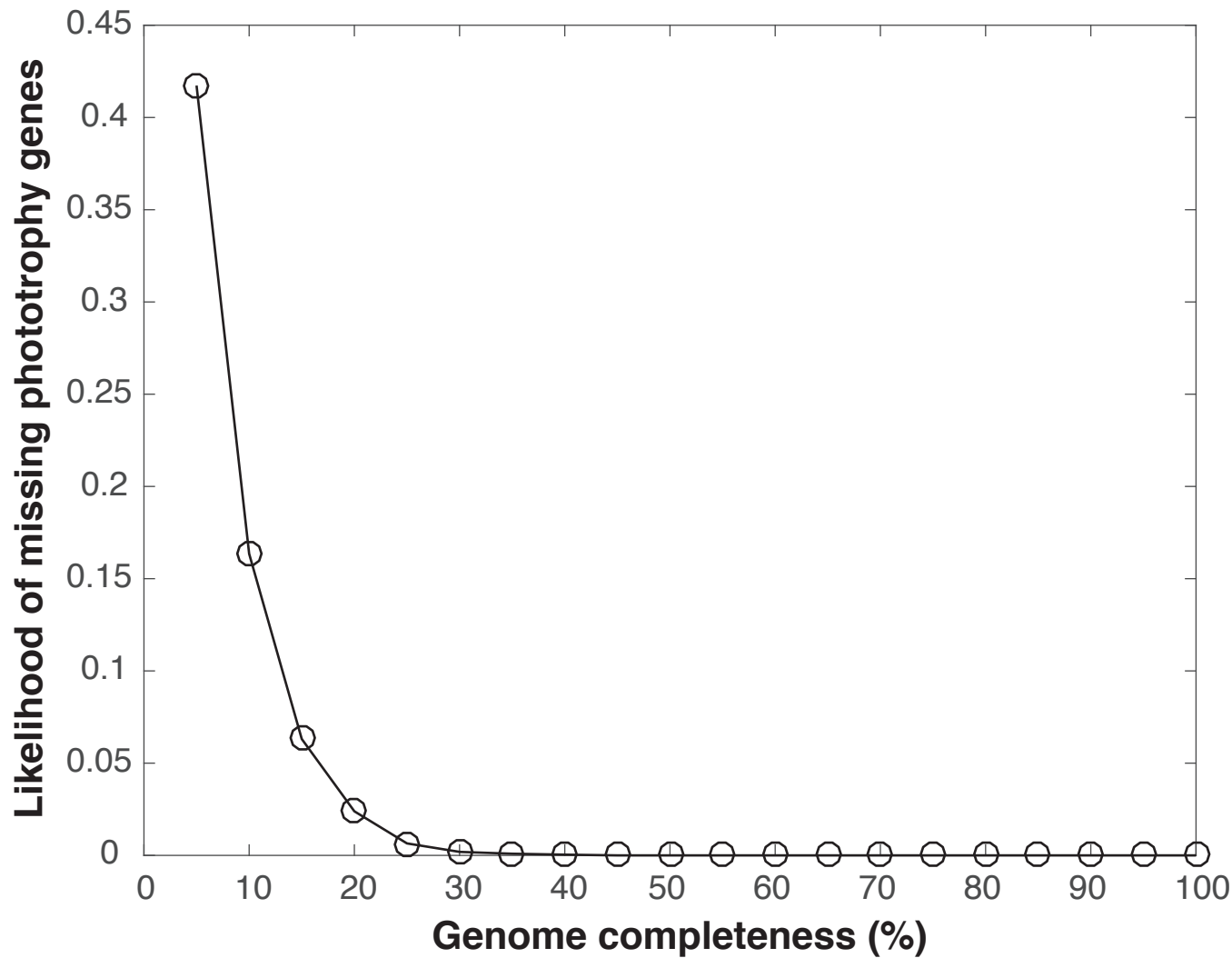

Supplement: Supplemental Figure 12 — “False negative” rate, or probability of failure to recover phototrophy genes, for a given completeness of genome recovery. Results plotted here are for a simulation following the constraints and logic discussed in the text. [file Image12.PDF]
