# Supplementary material for: Evolution of Phototrophy in the Chloroflexi Phylum Driven by Horizontal Gene Transfer
Source: Front Microbiol. 2018 Feb 19;9:260. doi: 10.3389/fmicb.2018.00260 (PMC5826079; doi:10.3389/fmicb.2018.00260)
Supplement: Supplemental Table 1 — Metagenome statistics. [file Table1.docx]

| ***Sample Name*** | ***Site*** | ***Raw Data*** | ***Assembly size*** | ***# Contigs*** | ***Largest contig*** | ***N50*** |
| --- | --- | --- | --- | --- | --- | --- |
| *CP1* | *Nakabusa* | *11.5 Gb* | *60 Mb* | *27594* | *147637* | *3053* |
| *CP2* | *Nakabusa* | *6.8 Gb* | *178 Mb* | *86054* | *724309* | *2507* |
| *JP1* | *Jinata* | *41.7 Gb* | *326 Mb* | *165282* | *234753* | *2221* |
| *JP3* | *Jinata* | *9.0 Gb* | *133 Mb* | *66077* | *219578* | *2465* |

Supplementary Table 1: Metagenome statistics
